# Global Trends in Death, Years of Life Lost, and Years Lived With Disability Caused by Breast Cancer Attributable to Secondhand Smoke From 1990 to 2019

**DOI:** 10.3389/fonc.2022.853038

**Published:** 2022-03-29

**Authors:** Zejin Ou, Yunxia Gao, Diwei Jiang, Jiaxin Cui, Yixian Ren, Shihao Tang, Danping Duan, Danfeng Yu, Zhi Wang

**Affiliations:** ^1^ Department of Central Laboratory, Guangzhou Twelfth People’s Hospital, Guangzhou, China; ^2^ Key Laboratory of Occupational Environment and Health, Guangzhou Twelfth People’s Hospital, Guangzhou, China; ^3^ School of Public Health, Guangzhou Medical University, Guangzhou, China; ^4^ School of Basic Medicine and Public Health, Jinan University, Guangzhou, China; ^5^ School of Public Health, Sun Yat-sen University, Guangzhou, China; ^6^ Department of Medical Intensive Care Unit (MICU), Guangdong Women and Children Hospital, Guangzhou, China

**Keywords:** breast cancer, secondhand smoke, global burden of disease, age-standardized rate, estimated annual percentage change

## Abstract

**Background:**

Secondhand smoke is an important risk factor to breast cancer patients’ survival. This article aimed to describe the epidemiological changes of health loss caused by female breast cancer attributable to secondhand smoke from 1990 to 2019.

**Methods:**

Data on breast cancer was derived from the Global Burden of Disease study 2019. The epidemiological status and trends were estimated using the number, age-standardized rate (ASR), and estimated annual percentage change (EAPC).

**Results:**

In 2019, secondhand smoke-related breast cancer caused 168.33×10^2^ death, 5242.58×10^2^ years of life lost (YLLs), and 334.03×10^2^ years lived with disability (YLDs) globally. The overall ASR of death and YLLs caused by breast cancer attributable to secondhand smoke presented decreasing trends from 1990 to 2019, with the respective EAPCs of −0.78 and −0.87. Meanwhile, decreasing trends occurred in most geographic regions, particularly that of YLLs in high-income North America (EAPC = −3.35). At the national level, most countries/territories had decreasing trends of death and YLLs, particularly Denmark, in which the respective EAPCs were −4.26 and −4.64. However, the ASR of YLDs showed an increasing trend globally (EAPC = 0.32). Meanwhile, increasing trends were observed in most regions and countries, particularly the Solomon Islands and Lesotho, with the respective EAPCs being 6.18 and 4.33. The changing trends were closely associated with sociodemographic development.

**Conclusions:**

Trends in secondhand smoke-related death and YLLs caused by breast cancer declined from 1990 to 2019. However, secondhand smoke remains a challenge to the patients’ longevity and quality of life. The findings informed strategies should be strengthened the control of secondhand smoking.

## Introduction

Breast cancer is the most common malignant tumor among women, and brought a substantial challenge to global health. The Global Burden of Disease study (GBDs) 2017 showed that breast cancer was the leading cause of cancer deaths and disability-adjusted life years (DALYs) for women, accounting for 601,000 deaths and 17.4 million DALYs (1). Among the related risk factors, secondhand smoke exposure is not only a well-demonstrated risk factor to the development of breast cancer (2–4), but also is an important influence factor of survival (5, 6). Globally, secondhand smoke caused more than additional 331,000 deaths and 9.3 million DALYs in 2013 (7). In Asia, secondhand smoke was a critical risk factor to DALYs caused by breast cancer (3.5%) (8). The proportion of secondhand smoke-related DALYs caused by breast cancer accounted for 1.0% in European Union (EU), and distributed heterogeneously across countries (9).

To achieve the goal of Sustainable Development Goals 2030, tobacco control had been implemented under the World Health Organization Framework Convention on Tobacco Control (WHO FCTC) since 2003 (10, 11). In recent years, the survival patterns of breast cancer and its related smoke exposure had changed (12, 13). The global age-standardized rate of death and DALYs caused by breast cancer attributable to secondhand smoke declined 5.4% and 4.9% during 2007-2017, respectively (13). However, serval countries reported the attributable YLLs of breast cancer improved slowly, substantially for tobacco exposure (14).

The GBDs accessed and quantified the burden of diseases, injuries, and risk factors, which facilitated tracking their epidemiological status and changes over time. By now, few studies reported the secondhand smoke-related health losses caused by breast cancer from a global landscape. Therefore, present work aimed to analyze health losses caused by breast cancer attributable to secondhand smoke worldwide, and estimate their trends with the updated GBDs data.

## Methods

### Data Source

Secondhand smoke is also called side-stream or passive smoke exposure. According to the instruction of GBDs, the definition of secondhand smoke exposure is that non-smokers are passively exposed to average daily particulate air matter from cigarette smoke with an aerodynamic diameter of smaller than 2.5 µg (measured in µg/m³) (7). Years of life lost (YLLs) and years lived with disability (YLDs) are the critical metrics of health loss reflecting the socioeconomic status and development of the health care system, both of which together are equal to disability-Adjusted Life-Years (DALYs). Data on secondhand smoke-related breast cancer was explored from the GBD study 2019 through the Global Health Data Exchange (GHDx) query tool (http://ghdx.healthdata.org/gbd-results-tool). The burden included death, YLLs, and YLDs, which were extracted for age groups, sociodemographic index (SDI) areas, geographic regions, and countries/territories from 1990 to 2019. An overview of the cancer burden was comprehensively presented globally, including 21 geographic regions and 204 countries/territories. The GBDs groups summarized and estimated the risks and exposures from 46,749 cohort studies, randomized controlled trials, civil surveys, and other sources, and more details on methods were seen in the previous studies (7, 13).

Sociodemographic index (SDI) is a compound index reflecting the influence of social development to civil health. The SDI value ranged from 0 to 1, which means the lowest and highest level of educational opportunities, average per capita incomes, and fertility rates. In 2019, the SDI scales varied from 0.081 in Somalia to 0.929 in Switzerland. According to the SDI, these countries/territories and regions were categorized into five levels, including low, low-middle, middle, high-middle, and high, with the respective upper bound of SDI quintiles being 0.454743, 0.607679, 0.689504, 0.805129, and 1.

### Statistical Analysis

The data involved different age structures in multiple populations over time, thus age-standardized is a necessary and representative index. The age-standardized rate (ASR) per 100,000 population was calculated using the following formula:


$$\text{ASR}=\frac{\Sigma_{i=1}^{A}a_{i}w_{i}}{\Sigma_{i=1}^{A}w_{i}}\times 100,000$$

Where *a_i_*: the age-specific rate in the *i* ^th^ age group; *w*: the number of the weight (people) in the corresponding *i* ^th^ age group among the selected standard population; *A*: the number of age groups.

Estimated annual percentage change (EAPC) is a widely used index to describe the epidemiological trends in the burden of diseases in public health studies (15, 16). EAPC could not only quantify the changing speed of ASR, but also estimate their future trends. A regression line is fitted to the natural logarithm of ASR, where y is the natural logarithm of ASR, and x is the calendar year. Subsequently, EAPC and its 95% confidence interval (CI) are estimated using the linear regression model. The formulas are presented as follows:


y=α+βx+ϵ

EAPC=100×(exp(β)−1)

The trends are judged as following standards: (1). if both the EAPC value and its 95% CI > 0, it is deemed to be an increasing trend of ASR; (2). if both the EAPC value and 95% CI < 0, it is deemed to be a decreasing trend of ASR; (3). others mean the ASR being stable over time. To analyze the influential factors of EAPC, the relationships between EAPCs and ASRs in 1990, and between ASRs and SDI in 2019 were calculated using a Pearson correlation analysis. The data and figures were analyzed using R v3.6.2 (Institute for Statistical Computing, Vienna, Austria). A *p-*value of less than 0.05 meant statistically significant.

## Results

### Trends of Death Caused by Breast Cancer Attributable to Secondhand Smoke

**Figure 1:**
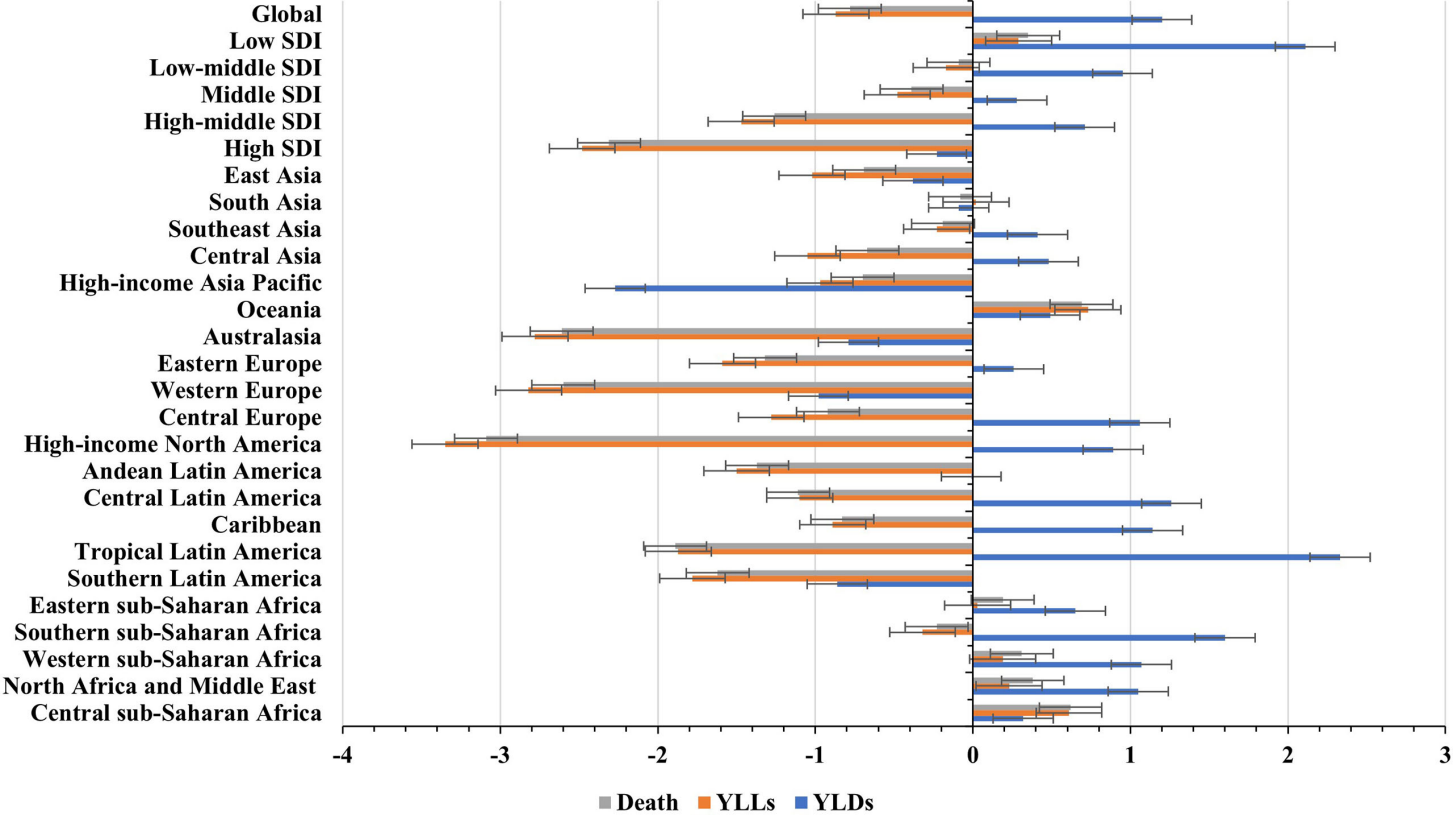
Global trends in the ASR of death, YLLs, and YLDs caused by breast cancer attributable to secondhand smoke, and in SDI areas and geographic regions from 1990 to 2019. ASR, age-standardized rate; SDI, sociodemographic index; YLLs, years of life lost; YLDs, years lived with disability.

**Figure 2:**
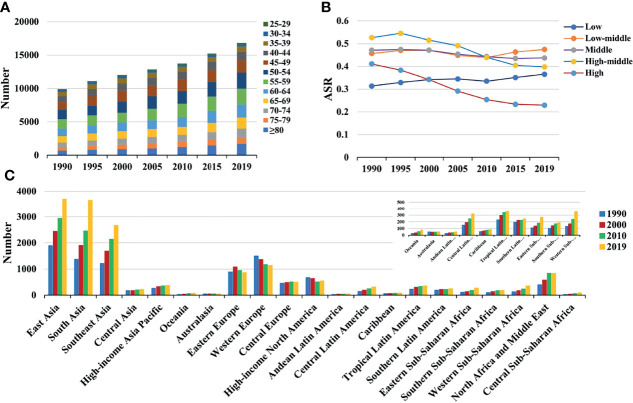
The distribution of death caused by breast cancer attributable to secondhand smoke from 1990 to 2019. **(A)** was the death number in age groups; **(B)** was the ASDR in SDI areas; **(C)** was the death number in geographical regions. ASDR, age-standardized death rate; SDI, sociodemographic index.

**Figure 3:**
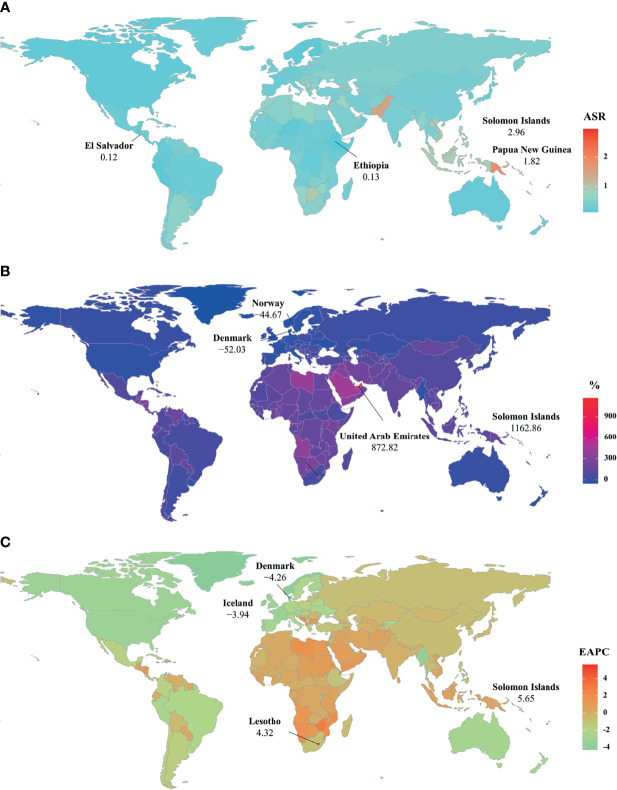
The distribution of ASRs, percentages, and EAPCs of death caused by breast cancer attributable to secondhand smoke at the national level. **(A)** was the ASDR in 2019; **(B)** was the percentage changes in death number between 2000 and 2019; **(C)** was the EAPCs of death, respectively. Countries/territories with an extreme value were annotated. ASDR, age-standardized death rate; EAPC, estimated annual percentage change.

Globally, breast cancer attributable to secondhand smoke was responsible for 168.33×10^2^ (95% uncertainty interval [UI]: 39.56×10^2^-290.39×10^2^) death in 2019, with an increase of 69.98% since 1990. The overall age-standardized death rate (ASDR) declined from 0.46 in 1990 to 0.39 in 2019, by an annual average decrease of 0.78% (EAPC = −0.78, 95%CI: −0.86 to −0.71) from 1990 to 2019 (**Table 1**; **Figure 1**). The age groups of 50-59 years undertook the most frequent of death cases in 2019, and all age groups had increasing percentages, particularly those aged above 80 years (145.85%) (**Supplementary Table 1**; **Figure 2A**). Regionally, the death number ranged from 0.48×10^2^ in Australasia to 37.00×10^2^ in East Asia in 2019. Trends of ASDR rose in five geographic regions, especially Oceania (EAPC = 0.69, 95%CI: 0.64-0.73). On the other hand, decreasing trends occurred in fifteen regions, and the most pronounced ones appeared in high-income North America (EAPC = −3.09, 95%CI: −3.29 to −2.88), followed by Australasia and tropical Latin America (**Table 1** **;** **Figures 1** and **2C**). Among 204 countries/territories, the burden of breast cancer attributable to secondhand smoke heterogeneously varied across countries. The ASDRs varied from 0.12 in El Salvador to 2.96 in the Solomon Islands in 2019. 1990-2019, the percentages of death number significantly increased in the Solomon Islands (1162.86%) and United Arab Emirates (872.82%), but pronouncedly decreased in Denmark (−52.03%), followed by Norway and Switzerland. Ninety-one countries undertook the increasing trends, particularly Solomon Islands and Lesotho, in which the respective EAPCs were 5.65 (95%CI: 5.02-6.29) and 4.32 (95%CI: 3.79-4.85). However, ninety-nine countries had decreasing trends, particularly Denmark and Iceland, in which the EAPCs were −4.26 (95%CI: −4.42 to −4.09) and −3.94 (95%CI: −4.14 to −3.73) (**Supplementary Table 2** **;** **Figures 3A** **–C**).

**Table 1:** the characteristics and trends of death caused by breast cancer attributable to secondhand smoke in global, SDI areas and geographic regions, 1990-2019.

Characteristics	1990		2019		1990-2019	
	Number×10^2^ (95% UI)	ASR/100,000 (95% UI)	Number×10^2^ (95% UI)	ASR/100,000 (95% UI)	Percentage (%)	EAPC (95%CI)
**Global**	99.03 (23.57–169.46)	0.46 (0.11–0.79)	168.33 (39.56-290.39)	0.39 (0.09-0.67)	69.98	−0.78 (−0.86-−0.71)
**SDI**						
Low	4.18 (1.02–7.38)	0.31 (0.08–0.55)	11.08 (2.54-19.59)	0.37 (0.08-0.65)	164.97	0.35 (0.26-0.45)
Low-middle	15.26 (3.68–26.48)	0.46 (0.11–0.80)	35.80 (8-61.94)	0.47 (0.11-0.82)	134.60	−0.09 (−0.20-0.03)
Middle	27.16 (6.41–46.65)	0.47 (0.11–0.81)	59.12 (14.48-102.13)	0.44 (0.11-0.76)	117.64	−0.39 (−0.44-−0.35)
High-middle	30.82 (7.38–52.86)	0.53 (0.13–0.90)	42.50 (10.26-73.46)	0.40 (0.10-0.69)	37.90	−1.26 (−1.39-−1.13)
High	21.55 (5.12–37.10)	0.41 (0.10–0.71)	19.72 (4.83-33.76)	0.23 (0.06-0.39)	−8.49	−2.31 (−2.43-−2.20)
**Regions**						
East Asia	18.95 (4.48–33.22)	0.40 (0.09–0.69)	37.00 (9.03-66.03)	0.34 (0.08-0.61)	95.23	−0.69 (−0.76-−0.63)
South Asia	13.65 (3.3–23.90)	0.45 (0.11–0.79)	36.52 (8.64-64.29)	0.48 (0.11-0.85)	167.52	−0.08 (−0.23-0.08)
Southeast Asia	12.12 (2.76–20.94)	0.77 (0.17–1.33)	26.63 (5.99-46.72)	0.75 (0.17-1.32)	119.83	−0.19 (−0.25-−0.12)
Central Asia	1.68 (0.41–2.87)	0.61 (0.15–1.04)	2.21 (0.53-3.82)	0.49 (0.12-0.85)	31.57	−0.67 (−0.78-−0.56)
High-income Asia Pacific	2.68 (0.63–4.57)	0.25 (0.06–0.42)	3.69 (0.88-6.37)	0.20 (0.05-0.35)	37.84	−0.70 (−0.80-−0.60)
Oceania	0.25 (0.06–0.45)	1.41 (0.33–2.54)	0.76 (0.18-1.38)	1.72 (0.41-3.14)	203.16	0.69 (0.64-0.73)
Australasia	0.49 (0.12–0.84)	0.42 (0.10–0.73)	0.48 (0.12-0.83)	0.21 (0.05-0.37)	−1.30	−2.61 (−2.77-−2.45)
Eastern Europe	8.92 (2.22–15.27)	0.55 (0.14–0.95)	8.60 (2.06-15.65)	0.45 (0.11-0.83)	−3.62	−1.32 (−1.67-−0.96)
Western Europe	14.92 (3.58–25.66)	0.54 (0.13–0.93)	11.29 (2.76-19.59)	0.27 (0.07-0.47)	−24.33	−2.60 (−2.69-−2.51)
Central Europe	4.52 (1.10–7.73)	0.57 (0.14–0.98)	4.97 (1.19-8.77)	0.45 (0.11-0.79)	9.96	−0.92 (−1.02-−0.83)
High-income North America	6.67 (1.6–11.59)	0.39 (0.09–0.68)	5.45 (1.31-9.41)	0.18 (0.04-0.31)	−18.35	−3.09 (−3.29-−2.88)
Andean Latin America	0.24 (0.05–0.42)	0.20 (0.05–0.36)	0.48 (0.11-0.88)	0.16 (0.04-0.29)	103.51	−1.37 (−1.57-−1.18)
Central Latin America	1.47 (0.36–2.53)	0.31 (0.08–0.53)	3.17 (0.77-5.59)	0.24 (0.06-0.43)	114.77	−1.11 (−1.26-−0.95)
Caribbean	0.51 (0.12–0.89)	0.37 (0.09–0.65)	0.82 (0.20-1.47)	0.30 (0.07-0.54)	59.77	−0.83 (−0.90-−0.76)
Tropical Latin America	2.29 (0.54–3.96)	0.45 (0.11–0.77)	3.56 (0.82-6.24)	0.27 (0.06-0.47)	55.18	−1.89 (−2.06-−1.72)
Southern Latin America	1.94 (0.46–3.35)	0.78 (0.18–1.34)	2.42 (0.57-4.21)	0.53 (0.12-0.92)	24.82	−1.62 (−1.74-−1.51)
Eastern Sub-Saharan Africa	1.08 (0.25–1.90)	0.25 (0.06–0.43)	2.68 (0.63-4.81)	0.27 (0.06-0.48)	148.34	0.19 (0.10-0.29)
Southern Sub-Saharan Africa	1.02 (0.25–1.79)	0.63 (0.16–1.11)	1.86 (0.45-3.29)	0.56 (0.14-0.99)	81.12	−0.23 (−0.36-−0.11)
Western Sub-Saharan Africa	1.30 (0.33–2.40)	0.28 (0.07–0.51)	3.53 (0.78-6.34)	0.31 (0.07-0.55)	170.66	0.31 (0.24-0.37)
North Africa and Middle East	4.02 (0.95–7.04)	0.42 (0.10–0.73)	11.33 (2.70-19.92)	0.47 (0.11-0.82)	181.51	0.38 (0.30-0.46)
Central Sub-Saharan Africa	0.29 (0.07–0.55)	0.21 (0.05–0.40)	0.90 (0.19-1.69)	0.26 (0.06-0.5)	204.78	0.62 (0.51-0.74)

EAPC, estimated annual percentage change; ASR, age-standardized rate; CI, confidence interval; UI, uncertainty interval; SDI, socio-demographic index.

### Trends of YLLs Caused by Breast Cancer Attributable to Secondhand Smoke

In 2019, an estimated 5242.58×10^2^ (95%UI: 1234.61×10^2^-9029.02×10^2^) YLLs number of breast cancer was attributable to secondhand smoke globally, and increased 60.83% since 1990. The overall ASR of YLLs presented a decreasing trend from 1990 to 2019, in which the EAPC was −0.87 (95%CI: −0.96 to −0.78) (**Table 2** **;** **Figure 1**). Those aged 50-54 years had the largest YLLs number (889.80×10^2^), and the percentages increased in all age groups, especially the patients above 80 years (135.52%) (**Supplementary Table 1** **;** **Supplementary Figure 1A**). Among 21 regions, the YLLs number ranged from 13.44×10^2^ in Australasia to 1214.48×10^2^ in South Asia in 2019. Decreasing trends in the ASR of YLLs occurred in 15 regions, and the largest one occurred in high-income North America (EAPC = −3.35, 95%CI: −3.58 to −3.13). Conversely, four regions showed increasing trends, including Oceania and Central Sub-Saharan Africa (**Table 2** **;** **Figure 1** and **Supplementary Figure 1C**). Nationally, the ASRs of YLLs caused by breast cancer attributable to secondhand smoke were heterogeneous across countries, ranging from 3.79 in El Salvador to 104.76 in Solomon Islands in 2019. 1990-2019, the percentage of YLLs number drastically increased in the Solomon Islands (1256.97%), but pronouncedly decreased in Denmark (−59.19%) and Norway (−48.16%). 107 countries/territories presented decreasing trends in the ASR of YLLs from 1990-2019, and Denmark had the most pronounced one (EAPC = −4.64, 95%CI: −4.82 to −4.46), followed by Norway and Myanmar. However, seventy-six countries showed increasing trends, particularly the Solomon Islands and Lesotho, in which the respective EAPCs were 6.01 (95%CI: 5.32-6.70) and 4.42 (95%CI: 3.85-4.99) (**Supplementary Table 3** **;** **Figure 3B** and **Supplementary Figures 2A–C**).

**Table 2:** the characteristics and trends of YLLs and YLDs caused by breast cancer attributable to secondhand smoke in global, SDI areas and geographic regions, 1990-2019.

Characteristics	YLLs				YLDs			
	2019		1990-2019		2019		1990-2019	
	Number×10^2^ (95% UI)	ASR/100,000(95% UI)	Percentage(%)	EAPC (95%CI)	Number×10^2^ (95% UI)	ASR/100,000(95% UI)	Percentage(%)	EAPC (95%CI)
**Global**	5242.58 (1234.61-9029.02)	12.28 (2.89-21.14)	60.83	−0.87 (−0.96-−0.78)	334.03 (74.04-602.13)	0.78 (0.17-1.41)	123.26	0.32 (0.28-0.36)
**SDI**								
Low	390.17 (90.65-692.44)	11.48 (2.64-20.27)	161.40	0.29 (0.19-0.39)	10.18 (2.34-19)	0.31 (0.07-0.58)	226.28	1.05 (0.98-1.13)
Low-middle	1193.14 (266.32-2063.49)	14.99 (3.35-25.89)	120.20	−0.17 (−0.30-−0.04)	40.88 (9.37-75.75)	0.52 (0.12-0.96)	216.27	1.07 (0.96-1.19)
Middle	1914.45 (469.83-3297.88)	13.76 (3.38-23.68)	101.78	−0.48 (−0.53-−0.42)	110.61 (24.3-200.67)	0.80 (0.18-1.45)	261.14	1.60 (1.56-1.63)
High-middle	1213.73 (294.18-2099.64)	11.94 (2.90-20.63)	25.45	−1.47 (−1.60-−1.33)	101.72 (22.47-184.5)	1.00 (0.22-1.81)	113.14	0.65 (0.58-0.72)
High	526.99 (129.86-903.45)	7.09 (1.75-12.24)	−18.96	−2.48 (−2.61-−2.36)	70.46 (15.31-130.46)	0.92 (0.20-1.72)	27.79	−0.86 (−0.97-−0.75)
**Regions**								
East Asia	1103.57 (266.85-1960.46)	10.29 (2.48-18.3)	70.24	−1.02 (−1.11-−0.92)	104.36 (22.86-193.17)	0.98 (0.21-1.81)	305.77	2.33 (2.25-2.40)
South Asia	1214.48 (288.49-2148.51)	15.10 (3.58-26.7)	154.26	0.02 (−0.14-0.18)	38.34 (8.91-70.88)	0.48 (0.11-0.89)	255.43	1.14 (0.99-1.28)
Southeast Asia	923.26 (208.25-1625.36)	24.96 (5.63-43.9)	106.51	−0.23 (−0.29-−0.16)	36.97 (8.59-69.19)	1.01 (0.23-1.89)	219.73	1.26 (1.22-1.29)
Central Asia	71.67 (17.13-123.55)	14.96 (3.6-25.76)	29.17	−1.05 (−1.17-−0.93)	3.60 (0.80-6.65)	0.76 (0.17-1.41)	69.32	−0.01 (−0.05-0.03)
High-income Asia Pacific	97.45 (23.58-165.61)	6.52 (1.59-11.01)	8.87	−0.97 (−1.10-−0.85)	15.42 (3.55-29.13)	0.98 (0.23-1.86)	82.39	0.89 (0.75-1.03)
Oceania	28.48 (7.04-51.62)	58.02 (14.07-105.51)	206.12	0.73 (0.68-0.78)	0.76 (0.17-1.45)	1.59 (0.36-3.02)	244.74	1.06 (1.02-1.11)
Australasia	13.44 (3.3-23.57)	6.63 (1.61-11.63)	−12.33	−2.78 (−2.95-−2.62)	1.90 (0.41-3.58)	0.92 (0.19-1.73)	48.62	−0.98 (−1.09-−0.87)
Eastern Europe	245.76 (59.23-446.97)	14.21 (3.44-25.78)	−12.30	−1.59 (−1.95-−1.23)	17.66 (3.9-33.37)	1.02 (0.23-1.9)	31.24	0.26 (0.10-0.41)
Western Europe	284.60 (70.58-490.9)	8.30 (2.07-14.39)	−34.36	−2.82 (−2.92-−2.73)	37.75 (8.12-70.2)	1.09 (0.24-2.04)	12.61	−0.79 (−0.94-−0.64)
Central Europe	117.66 (28.28-209.62)	12.17 (2.93-21.72)	−9.82	−1.28 (−1.37-−1.19)	9.37 (2.08-17.39)	0.97 (0.21-1.81)	42.53	0.49 (0.37-0.6)
High-income North America	144.73 (35.04-251.18)	5.47 (1.32-9.52)	−28.67	−3.35 (−3.58-−3.13)	21.13 (4.24-40.13)	0.77 (0.15-1.47)	0.69	−2.27 (−2.46-−2.07)
Andean Latin America	15.25 (3.59-28.02)	4.93 (1.16-9.06)	86.87	−1.50 (−1.69-−1.31)	0.73 (0.16-1.4)	0.24 (0.05-0.46)	234.25	0.48 (0.32-0.64)
Central Latin America	98.99 (23.93-174.6)	7.40 (1.79-13.07)	99.02	−1.10 (−1.25-−0.95)	6.02 (1.36-11.47)	0.45 (0.1-0.86)	227.12	0.41 (0.27-0.55)
Caribbean	24.22 (5.70-44.03)	9.09 (2.14-16.56)	49.11	−0.89 (−0.96-−0.81)	1.40 (0.31-2.58)	0.52 (0.11-0.96)	90.42	−0.09 (−0.13-−0.06)
Tropical Latin America	105.04 (23.89-184.41)	7.82 (1.78-13.7)	42.65	−1.87 (−2.03-−1.71)	5.77 (1.31-10.93)	0.43 (0.10-0.81)	127.98	−0.38 (−0.60-−0.16)
Southern Latin America	57.86 (13.71-100.98)	13.62 (3.23-23.7)	10.83	−1.78 (−1.88-−1.67)	3.82 (0.80-7.32)	0.89 (0.19-1.72)	73.33	−0.23 (−0.33-−0.12)
Eastern Sub-Saharan Africa	95.15 (22.67-171.11)	8.28 (1.95-14.89)	142.92	0.03 (−0.07-0.14)	2.40 (0.54-4.60)	0.22 (0.05-0.41)	199.99	0.71 (0.62-0.81)
Southern Sub-Saharan Africa	56.12 (13.38-99.37)	15.69 (3.77-27.7)	68.29	−0.32 (−0.46-−0.17)	1.87 (0.43-3.49)	0.53 (0.12-0.99)	103.56	0.28 (0.17-0.39)
Western Sub-Saharan Africa	123.35 (27.15-223.57)	9.30 (2.05-16.72)	176.59	0.19 (0.12-0.27)	3.32 (0.72-6.46)	0.26 (0.06-0.51)	238.93	0.95 (0.89-1.01)
North Africa and Middle East	389.44 (92.71-689)	14.70 (3.5-25.93)	173.73	0.23 (0.14-0.31)	20.68 (4.66-38.67)	0.79 (0.18-1.48)	360.23	2.11 (2.04-2.18)
Central Sub-Saharan Africa	32.05 (6.71-60.26)	8.27 (1.74-15.63)	201.79	0.61 (0.51-0.71)	0.76 (0.17-1.51)	0.20 (0.05-0.40)	260.33	1.20 (1.06-1.33)

EAPC, estimated annual percentage change; ASR, age-standardized rate; CI, confidence interval; UI, uncertainty interval; SDI, socio-demographic index; YLLs, years of life lost; YLDs, years lived with disability.

### Trends of YLDs Caused by Breast Cancer Attributable to Secondhand Smoke

Globally, the number of YLDs caused by breast cancer attributable to secondhand smoke was 334.03×10^2^ (95%UI: 74.04×10^2^-602.13×10^2^) in 2019, by an increasing percentage of 123.26% since 1990. The global ASR of YLDs increased with an annual average 0.32% from 1990 to 2019 (EAPC = 0.32, 95%CI: 0.28-0.36) (**Table 2** **;** **Figure 1**). The highest YLDs number was seen in those aged 50-54 years in 2019 (51.56×10^2^), and the largest increasing percentages occurred in the people above 80 years (155.79%) **(** **Supplementary Table 1** **;** **Supplementary Figure 3A**). Among 21 geographic regions, the YLDs number ranged from 0.73×10^2^ in Andean Latin America to 104.36×10^2^ in East Asia in 2019. Fourteen regions appeared increasing trends in the ASR of YLDs, and East Asia had the largest one (EAPC = 2.33, 95%CI: 2.25-2.40), followed by North Africa and Middle East and Southeast Asia. Whereas six regions had decreasing trends, particularly high-income North America (EAPC = −2.27, 95%CI: −2.46 to −2.07) (**Table 2** **;** **Figure 1** and **Supplementary Figure 3C**). Among 204 countries/territories, the ASR of YLDs ranged from 0.11 in Ethiopia to 3.01 in Solomon Islands in 2019. 1990-2019, pronounced increasing percentages were seen in Solomon Islands (1424.03%) and United Arab Emirates (1351.36%). Whereas Denmark had the largest decreasing one (−22.79%), followed by Ukraine and Georgia. 145 countries/territories showed increasing trends in ASR of YLDs caused by breast cancer attributable to secondhand smoke over the past three decades, and the Solomon Islands had the most pronounced ones (EAPC = 6.18, 95%CI: 5.66-6.70), followed by Lesotho and Saudi Arabia. On the other hand, only forty-eight countries had decreasing trends, particularly Iceland and Myanmar, in which the respective EAPCs were −2.56 (95%CI: −2.70 to −2.42) and −2.46 (95%CI: −2.71 to −2.21) (**Supplementary Table 4**; **Supplementary Figures 4A–C**).

### The Burden of Breast Cancer Attributable to Secondhand Smoke-Related With SDI

**Figure 4:**
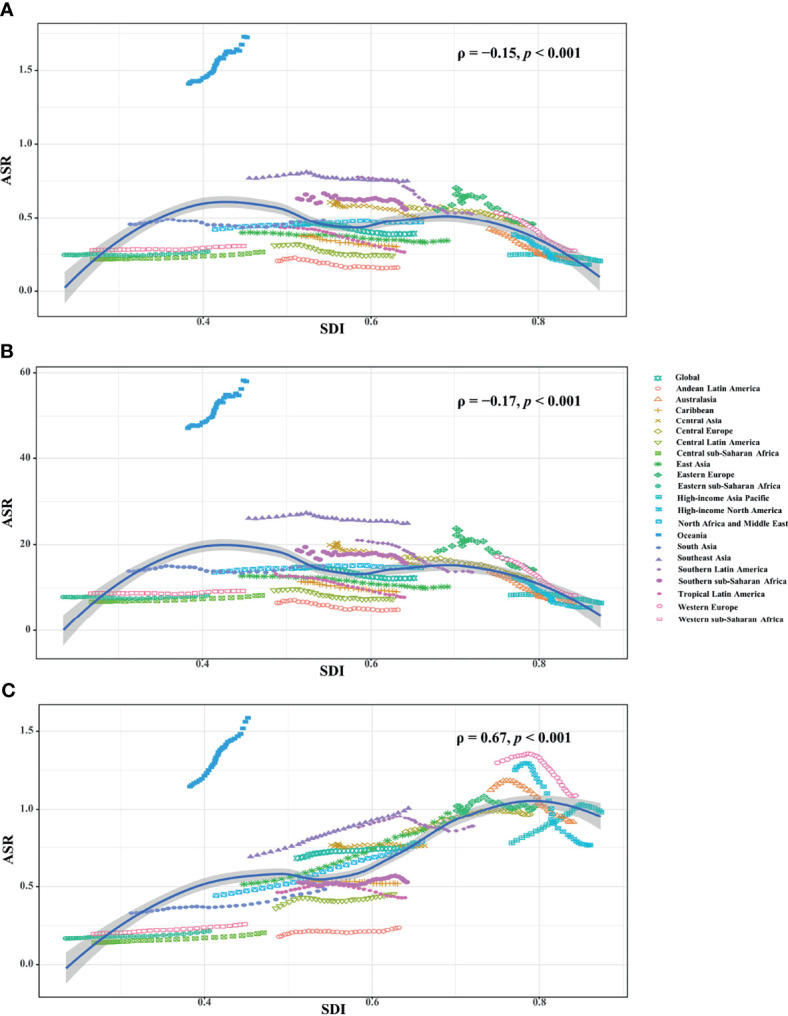
Correlation analysis between ASRs and SDI in 2019 among regions. The ASRs of death **(A)** and YLLs **(B)** negatively related with SDI, but that of YLDs **(C)** positively related with SDI. The correlations were calculated with Pearson correlation analysis. ASR, age-standardized rate; SDI, socio-demographic index; YLLs, years of life lost; YLDs, years lived with disability.

Among five SDI areas, the middle SDI area had the largest health loss caused by breast cancer attributable to secondhand smoke, followed by low-middle and high-middle areas. The ASDR ranged from 0.23 in the high SDI area to 0.47 in the low-middle one.1990-2019, decreasing trends in the ASR of death and YLLs caused by breast cancer attributable to secondhand smoke occurred in most SDI areas, particularly high SDI one, with the respective EAPCs being −2.31 (95%CI: −2.43 to −2.20) and −2.48 (95%CI:−2.61 to −2.36). However, increasing trends of death and YLLs appeared in the low SDI area. On the other hand, increasing trends of YLDs occurred in most SDI areas, except the high SDI one (EAPC = −0.86, 95%CI: −0.97 to −0.75). The most pronounced increasing one was seen in the middle SDI area (EAPC = 1.60, 95%CI: 1.56 to 1.63), followed by low and low-middle SDI ones. (**Tables 1**, **2** **;** **Figures 1**, **2B**). Negative correlations were found between ASRs of death and YLLs and SDI among regions in 2019. Whereas positive correlation was found between ASRs of YLDs and SDI (ρ = −0.15, *p* < 0.001; ρ = −0.17, *p* < 0.001; ρ = 0.67, *p* < 0.001, respectively; **Figures 4A** **–C**).

**Figure 5:**
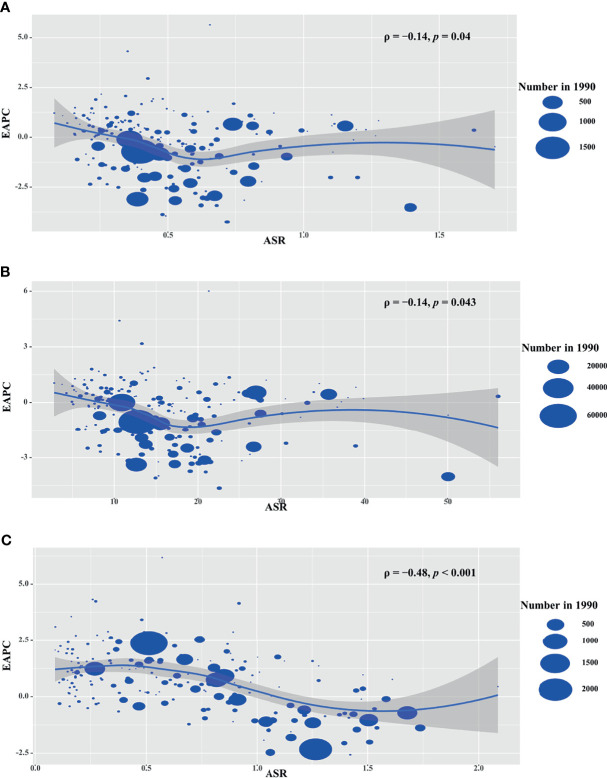
Correlation analysis between EAPCs and ASRs in 1990 among countries. The ASRs of death **(A)**, YLLs **(B)**, and YLDs **(C)** had negative correlations with ASR, respectively. The correlations were calculated with Pearson correlation analysis. The symbols were the countries/territories in the corresponding regions. ASR, age-standardized rate; YLLs, years of life lost; YLDs, years lived with disability.

The ASRs in 1990 is considered as the disease reservoir at baseline. EAPCs of death, YLLs and YLDs had negative relationships with their corresponding ASRs in 1990 at the national level (ρ = −0.14, p = 0.04; ρ = −0.14, p = 0.043; ρ = −0.48, p < 0.001, respectively; **Figures 5A** **–C**).

## Discussion

Secondhand smoke is an important risk factor to the development and survival of breast cancer. Based on the pooled analysis, both passive and active smoking were demonstrated to equally elevate the risk of breast cancer for women (17). It was estimated that passive smoke exposure caused two-fold breast cancer mortality among never smokers (Hazard ratios = 2.12, 95% CI = 1.24-3.63) (6). The potential mechanisms included the changes in DNA methylation (18), hormone-receptor status (19), genetic susceptibility (20), and hormone levels (particularly premenopausal) (21). Meanwhile, passive smoke exposure probably stimulated the malignant performance of cancer, including cell malignancy, tumor angiogenesis, and metastatic activity (22–25).

Despite the overall incidence of breast cancer growing steadily, improvements in the survival of the patients had been achieved over the past decades (12, 26). The findings in the present work showed the decreasing trend in the death and YLLs caused by breast cancer attributable to secondhand smoke during the period 1990-2019. The achievements benefited from the early screening, improved regimens and healthcare systems, and the management of related risk factors (1, 12, 27). Meanwhile, a large reduction in the prevalence of daily smoke exposure had been achieved globally (28, 29), particularly cost-effective smoke-free policies for reducing secondhand smoke exposure under the forced measures of WHO FCTC (30, 31). Additionally, decreasing trends in secondhand smoke exposure were observed during 2011-2018 (32), and it was estimated that 1 in 14 breast cancer cases could be prevented without secondhand smoke exposure (33). Sociodemographic status strongly influenced the prevalence of smoking and second-hand smoke exposure (34). The upward trends of YLDs caused by breast cancer attributable to secondhand smoke were seen globally, and in most regions and countries. The improved patients’ survival could prolong the lifespan with disability. Passive smoke had a high risk of early-onset breast cancer, probably related to genetic polymorphisms (35).

Among SDI quintiles, the middle and high-middle SDI areas undertook the largest burden caused by breast cancer attributable to secondhand smoke, mainly related with the huge population size, and rapid population growth and aging (1, 7). Meanwhile, several low-income and middle-income countries faced the challenge of worsening smoke epidemics (36). Because of poor policy guidance and health awareness, cigarette consumption remains to be unfavorable in low- and middle-income countries (37). Furthermore, the poor medical resource and healthcare system drove the increasing trends in the low SDI area (38). At the national level, downward trends of death and YLLs commonly occurred in the high SDI countries, particularly the high-income North America and Western Europe, where existed sound health systems, early disease screening, and strict tobacco control policies. In high-income North America, the implementation of home smoking bans had significantly reduced the exposure of secondhand smoke at home (39). Denmark, Iceland, and Norway presented the most pronounced decreasing trends, were the highest score of the implementation of tobacco control policies (40). On the other hand, increasing trends were mostly seen in the south Pacific and sub-Saharan Africa countries, where the new diagnosed cases of breast cancer were much more than in the past, lack of cancer prevention and control programs (41, 42), low awareness of cancer risk, and weak implementation of smoke-free policies in public places (43, 44).

Several limitations should be interpreted in this work. First, the GBD estimations of passive smoking exposure were mainly based on the data from multiple sources, including cross-sectional survey, self-reported data, history recall, and so on. The potential bias was inevitable, including underreported cases, incomplete testing, and the technology varied across countries over time (7, 13, 38). Second, data on the intensity of secondhand smoke exposure could not be quantified and categorized, thus analysis on the association between the exposure and health loss. Third, individual heterogeneity was prone to different subtypes of breast cancer, including genetic background, hormone, and physiological status (pre/postmenopausal). However, the lack of related data failed to explain the findings further. Fourth, several countries lack vital registration data, but the GBD collaborators applied multi statistical models to estimate the settings with sparse data. Last but not least, age is an important factor in breast cancer. However, due to the limitation of ASR estimates, the trends were demonstrated only using the percentage changes in the absolute number in age groups.

## Conclusions

1990-2019, trends of secondhand smoke-related death and YLLs due to breast cancer declined worldwide, and in most regions and countries, highlighting that the advance in the current management and treatment of the disease. Healthcare systems need to be improved to cope with the increasing trends in disability caused by the disease. Meanwhile, disparities and inequities of health care existed among regions and countries, suggesting global efforts need to be taken to promote health equity. Secondhand smoke exposure is a modifiable risk factor, and governments should adopt cost-effective measures to reduce the related burden through the implementation of enhanced smoke-free policies in public places.

## Data Availability Statement

The original contributions presented in the study are included in the article/**Supplementary Material**. Further inquiries can be directed to the corresponding authors.

## Author Contributions

ZO: Project administration and drafting. DJ, ST, and YR: Data analysis and validation. DY and YG: Data analysis and visualization. JC and DD: Data collection and collation. ZW: supervision and drafting and editing. All authors contributed to the article and approved the submitted version.

## Funding

Guangzhou Health Science and Technology Major Project (No.:2021A031003).

## Conflict of Interest

The authors declare that the research was conducted in the absence of any commercial or financial relationships that could be construed as a potential conflict of interest.

## Publisher’s Note

All claims expressed in this article are solely those of the authors and do not necessarily represent those of their affiliated organizations, or those of the publisher, the editors and the reviewers. Any product that may be evaluated in this article, or claim that may be made by its manufacturer, is not guaranteed or endorsed by the publisher.

## Abbreviations

GBD, Global Burden of Disease; YLLs, years of life lost; YLDs, years lived with disability; DALYs, Disability-adjusted life years; ASR, Age-standardized rate; UI, Uncertainty interval; CI, Confidence interval; EAPC, Estimated annual percentage change; GHDx, Global Health Data Exchange; SDI, Socio-demographic index; WHO FCTC, the World Health Organization Framework Convention on Tobacco Control.

## References

[1] Global Burden of Disease Cancer Collaboration Fitzmaurice C Abate D Abbasi N Moghadam TZ Zendehdel K. Global, Regional, and National Cancer Incidence, Mortality, Years of Life Lost, Years Lived With Disability, and Disability-Adjusted Life-Years for 29 Cancer Groups, 1990 to 2017: A Systematic Analysis for the Global Burden of Disease Study. JAMA Oncol (2019) 5(12):1749–69. doi: 10.1001/jamaoncol.2019.2996 PMC6777271 31560378

[2] Chen C Huang YB Liu XO Gao Y Dai HJ Song FJ. Active and Passive Smoking With Breast Cancer Risk for Chinese Females: A Systematic Review and Meta-Analysis. Chin J Cancer (2014) 33(6):306–16. doi: 10.5732/cjc.013.10248 PMC4059868 24823992

[3] Dossus L Boutron-Ruault MC Kaaks R Gram IT Vilier A Fervers B. Active and Passive Cigarette Smoking and Breast Cancer Risk: Results From the EPIC Cohort. Int J Cancer (2014) 134(8):1871–88. doi: 10.1002/ijc.28508 24590452

[4] Kim AS Ko HJ Kwon JH Lee JM. Exposure to Secondhand Smoke and Risk of Cancer in Never Smokers: A Meta-Analysis of Epidemiologic Studies. Int J Environ Res Public Health (2018) 15(9):1981–98. doi: 10.3390/ijerph15091981 PMC6164459 30208628

[5] Islami F Goding Sauer A Miller KD Siegel RL Fedewa SA Jacobs EJ. Proportion and Number of Cancer Cases and Deaths Attributable to Potentially Modifiable Risk Factors in the United States. CA Cancer J Clin (2018) 68(1):31–54. doi: 10.3322/caac.21440 29160902

[6] Boone SD Baumgartner KB Baumgartner RN Connor AE John EM Giuliano AR. Active and Passive Cigarette Smoking and Mortality Among Hispanic and non-Hispanic White Women Diagnosed With Invasive Breast Cancer. Ann Epidemiol (2015) 25(11):824–31. doi: 10.1016/j.annepidem.2015.08.007 PMC4609618 26387598

[7] GBD Risk Factors Collaborators Forouzanfar MH Alexander L Anderson HR Bachman VF Lopez AD. Global, Regional, and National Comparative Risk Assessment of 79 Behavioural, Environmental and Occupational, and Metabolic Risks or Clusters of Risks in 188 Countries, 1990-2013: A Systematic Analysis for the Global Burden of Disease Study 2013. Lancet (2015) 386(10010):2287–323. doi: 10.1016/S0140-6736(15)00128-2 PMC4685753 26364544

[8] Sharma R. Examination of Incidence, Mortality and Disability-Adjusted Life Years and Risk Factors of Breast Cancer in 49 Asian Countries, 1990-2019: Estimates From Global Burden of Disease Study 2019. Jpn J Clin Oncol (2021) 51(5):1927–35. doi: 10.1093/jjco/hyab004 33621341

[9] Carreras G Lachi A Boffi R Clancy L Gallus S Fernandez E. Burden of Disease From Breast Cancer Attributable to Smoking and Second-Hand Smoke Exposure in Europe. Int J Cancer (2020) 147(9):2387–93. doi: 10.1002/ijc.33021 32356370

[10] Burci GL. World Health Organization (WHO): Framework Convention on Tobacco Control. International Legal Materials, Vol. 42. Cambridge, UK: Cambridge University Press (2003). pp. 515–39. doi: 10.1017/S0020782900010202.

[11] Fong GT Yuan J Craig LV Xu SS Meng G Quah ACK. Achieving the Goals of Healthy China 2030 Depends on Increasing Smoking Cessation in China: Comparative Findings From the ITC Project in China, Japan, and the Republic of Korea. China CDC Wkly (2021) 3(22):463–7. doi: 10.46234/ccdcw2021.120 PMC8393109 34594914

[12] Li N Deng Y Zhou L Tian T Yang S Wu Y. Global Burden of Breast Cancer and Attributable Risk Factors in 195 Countries and Territories, From 1990 to 2017: Results From the Global Burden of Disease Study 2017. J Hematol Oncol (2019) 12(1):140. doi: 10.1186/s13045-019-0828-0 31864424 PMC6925497

[13] GBD Risk Factor Collaborators. Global, Regional, and National Comparative Risk Assessment of 84 Behavioural, Environmental and Occupational, and Metabolic Risks or Clusters of Risks for 195 Countries and Territories, 1990-2017: A Systematic Analysis for the Global Burden of Disease Study 2017. Lancet (2018) 392(10159):1923–94. doi: 10.1016/S0140-6736(18)32225-6 PMC6227755 30496105

[14] Steel N Ford JA Newton JN Davis ACJ Vos T Naghavi M. Changes in Health in the Countries of the UK and 150 English Local Authority Areas 1990-2016: A Systematic Analysis for the Global Burden of Disease Study 2016. Lancet (2018) 392(10158):1647–61. doi: 10.1016/S0140-6736(18)32207-4 PMC6215773 30497795

[15] Ou ZJ Yu DF Liang YH He WQ Li YZ Meng YX. Trends in Burden of Multidrug-Resistant Tuberculosis in Countries, Regions, and Worldwide From 1990 to 2017: Results From the Global Burden of Disease Study. Infect Dis Poverty (2021) 10(1):24. doi: 10.1186/s40249-021-00803-w 33676581 PMC7936417

[16] Hankey BF Ries LA Kosary CL Feuer EJ Merrill RM Clegg LX. Partitioning Linear Trends in Age-Adjusted Rates. Cancer Causes Control (2000) 11(1):31–5. doi: 10.1023/a:1008953201688 10680727

[17] Sadri G Mahjub H. Passive or Active Smoking, Which Is More Relevant to Breast Cancer. Saudi Med J (2007) 28(2):254–8. doi: 10.1016/j.revmed.2006.10.323 17268706

[18] Callahan CL Bonner MR Nie J Wang Y Tao MH Shields PG. Active and Secondhand Smoke Exposure Throughout Life and DNA Methylation in Breast Tumors. Cancer Causes Control (2019) 30(1):53–62. doi: 10.1007/s10552-018-1102-4 30617699 PMC6697076

[19] Tong JH Li Z Shi J Li HM Wang Y Fu LY. Passive Smoking Exposure From Partners as a Risk Factor for ER+/PR+ Double Positive Breast Cancer in Never-Smoking Chinese Urban Women: A Hospital-Based Matched Case Control Study. PloS One (2014) 9(5):e97498. doi: 10.1371/journal.pone.0097498 24866166 PMC4035255

[20] Slattery ML Curtin K Giuliano AR Sweeney C Baumgartner R Edwards S. Active and Passive Smoking, IL6, ESR1, and Breast Cancer Risk. Breast Cancer Res Treat (2008) 109(1):101–11. doi: 10.1007/s10549-007-9629-1 PMC2532584 17594514

[21] Hanaoka T Yamamoto S Sobue T Sasaki S Tsugane S JPHC-BPSo C. Active and Passive Smoking and Breast Cancer Risk in Middle-Aged Japanese Women. Int J Cancer (2005) 114(2):317–22. doi: 10.1002/ijc.20709 15540214

[22] Daniell HW. Increased Lymph Node Metastases at Mastectomy for Breast Cancer Associated With Host Obesity, Cigarette Smoking, Age, and Large Tumor Size. Cancer (1988) 62(2):429–35. doi: 10.1002/1097-0142(19880715)62:2<429::aid-cncr2820620230>3.0.co;2-4 3383142

[23] Kobrinsky NL Klug MG Hokanson PJ Sjolander DE Burd L. Impact of Smoking on Cancer Stage at Diagnosis. J Clin Oncol (2003) 21(5):907–13. doi: 10.1200/JCO.2003.05.110 12610192

[24] Maclure M Katz RB Bryant MS Skipper PL Tannenbaum SR. Elevated Blood Levels of Carcinogens in Passive Smokers. Am J Public Health (1989) 79(10):1381–4. doi: 10.2105/ajph.79.10.1381 PMC1350179 2782507

[25] Jang S Prizment A Haddad T Robien K Lazovich D. Smoking and Quality of Life Among Female Survivors of Breast, Colorectal and Endometrial Cancers in a Prospective Cohort Study. J Cancer Surviv (2011) 5(2):115–22. doi: 10.1007/s11764-010-0147-5 PMC4093827 20812033

[26] Global Burden of Disease Cancer Collaboration Fitzmaurice C Akinyemiju TF Al Lami FH Alam T Alizadeh-Navaei R. Global, Regional, and National Cancer Incidence, Mortality, Years of Life Lost, Years Lived With Disability, and Disability-Adjusted Life-Years for 29 Cancer Groups, 1990 to 2016: A Systematic Analysis for the Global Burden of Disease Study. JAMA Oncol (2018) 4(11):1553–68. doi: 10.1001/jamaoncol.2018.2706 PMC6248091 29860482

[27] Bray F Ferlay J Soerjomataram I Siegel RL Torre LA Jemal A. Global Cancer Statistics 2018: GLOBOCAN Estimates of Incidence and Mortality Worldwide for 36 Cancers in 185 Countries. CA Cancer J Clin (2018) 68(6):394–424. doi: 10.3322/caac.21492 30207593

[28] Ng M Freeman MK Fleming TD Robinson M Dwyer-Lindgren L Thomson B. Smoking Prevalence and Cigarette Consumption in 187 Countries, 1980-2012. JAMA (2014) 311(2):183–92. doi: 10.1001/jama.2013.284692 24399557

[29] Flor LS Reitsma MB Gupta V Ng M Gakidou E. The Effects of Tobacco Control Policies on Global Smoking Prevalence. Nat Med (2021) 27(2):239–43. doi: 10.1038/s41591-020-01210-8 PMC7884287 33479500

[30] Gravely S Giovino GA Craig L Commar A D'Espaignet ET Schotte K. Implementation of Key Demand-Reduction Measures of the WHO Framework Convention on Tobacco Control and Change in Smoking Prevalence in 126 Countries: An Association Study. Lancet Public Health (2017) 2(4):e166–e74. doi: 10.1016/S2468-2667(17)30045-2 29253448

[31] Ngo A Cheng KW Chaloupka FJ Shang C. The Effect of MPOWER Scores on Cigarette Smoking Prevalence and Consumption. Prev Med (2017) 105S:S10–S4. doi: 10.1016/jypmed.2017.05.006 PMC5681881 28502576

[32] Tsai J Homa DM Neff LJ Sosnoff CS Wang L Blount BC. Trends in Secondhand Smoke Exposure, 2011-2018: Impact and Implications of Expanding Serum Cotinine Range. Am J Prev Med (2021) 61(3):e109-e17. doi: 10.1016/j.amepre.2021.04.004 34419235

[33] Gram IT Wiik AB Lund E Licaj I Braaten T. Never-Smokers and the Fraction of Breast Cancer Attributable to Second-Hand Smoke From Parents During Childhood: The Norwegian Women and Cancer Study 1991-2018. Int J Epidemiol (2022) 50(6):1927–35. doi: 10.1093/ije/dyab153 PMC8743119 34999865

[34] Zheng Y Ji Y Dong H Chang C. The Prevalence of Smoking, Second-Hand Smoke Exposure, and Knowledge of the Health Hazards of Smoking Among Internal Migrants in 12 Provinces in China: A Cross-Sectional Analysis. BMC Public Health (2018) 18(1):655. doi: 10.1186/s12889-018-5549-8 29793454 PMC5968586

[35] Daly AA Rolph R Cutress RI Copson ER. A Review of Modifiable Risk Factors in Young Women for the Prevention of Breast Cancer. Breast Cancer (Dove Med Press) (2021) 13:241–57. doi: 10.2147/BCTT.S268401 PMC8053601 33883932

[36] Bilano V Gilmour S Moffiet T d'Espaignet ET Stevens GA Commar A. Global Trends and Projections for Tobacco Use, 1990-2025: An Analysis of Smoking Indicators From the WHO Comprehensive Information Systems for Tobacco Control. Lancet (2015) 385(9972):966–76. doi: 10.1016/S0140-6736(15)60264-1 25784347

[37] Anderson CL Becher H Winkler V. Tobacco Control Progress in Low and Middle Income Countries in Comparison to High Income Countries. Int J Environ Res Public Health (2016) 13(10):1039–53. doi: 10.3390/ijerph13101039 PMC5086778 27783060

[38] GBD Tobacco Collaborators. Smoking Prevalence and Attributable Disease Burden in 195 Countries and Territories, 1990-2015: A Systematic Analysis From the Global Burden of Disease Study 2015. Lancet (2017) 389(10082):1885–906. doi: 10.1016/S0140-6736(17)30819-X PMC5439023 28390697

[39] Mills AL White MM Pierce JP Messer K. Home Smoking Bans Among U.S. Households With Children and Smokers. Opportunities for Intervention. Am J Prev Med (2011) 41(6):559–65. doi: 10.1016/j.amepre.2011.08.016 22099231

[40] Joossens L Raw M. The Tobacco Control Scale: A New Scale to Measure Country Activity. Tob Control (2006) 15(3):247–53. doi: 10.1136/tc.2005.015347 PMC2564668 16728757

[41] Moore MA Baumann F Foliaki S Goodman MT Haddock R Maraka R. Cancer Epidemiology in the Pacific Islands - Past, Present and Future. Asian Pac J Cancer Prev (2010) 11(Suppl 2):99–106. doi: 10.1097/CAD.0b013e328335be46 20553071 PMC4386924

[42] Jemal A Bray F Forman D O'Brien M Ferlay J Center M. Cancer Burden in Africa and Opportunities for Prevention. Cancer (2012) 118(18):4372–84. doi: 10.1002/cncr.27410 22252462

[43] Mamudu HM Owusu D Asare B Williams F Asare M Oke A. Support for Smoke-Free Public Places Among Adults in Four Countries in Sub-Saharan Africa. Nicotine Tob Res (2020) 22(12):2141–8. doi: 10.1093/ntr/ntaa008 PMC7733060 31927591

[44] Ayo-Yusuf OA Olufajo O Agaku IT. Exposure to Secondhand Smoke and Voluntary Adoption of Smoke-Free Home and Car Rules Among Non-Smoking South African Adults. BMC Public Health (2014) 14:580. doi: 10.1186/1471-2458-14-580 24913038 PMC4064503

