# Supplementary material for: Global Trends in Death, Years of Life Lost, and Years Lived With Disability Caused by Breast Cancer Attributable to Secondhand Smoke From 1990 to 2019
Source: Front Oncol. 2022 Mar 29;12:853038. doi: 10.3389/fonc.2022.853038 (PMC9001985; doi:10.3389/fonc.2022.853038)
Supplement: Supplementary file 1 [file DataSheet_1.docx]

**Supplementary figure 1**. The distribution of YLLs caused by breast cancer attributable to secondhand smoke from 1990 to 2019. (A) was the number of YLLs in age groups; (B) was the ASR of YLLs in SDI areas; (C) was the number of YLLs in geographical regions. ASR, age-standardized rate; SDI, sociodemographic index; YLLs, years of life lost.

**
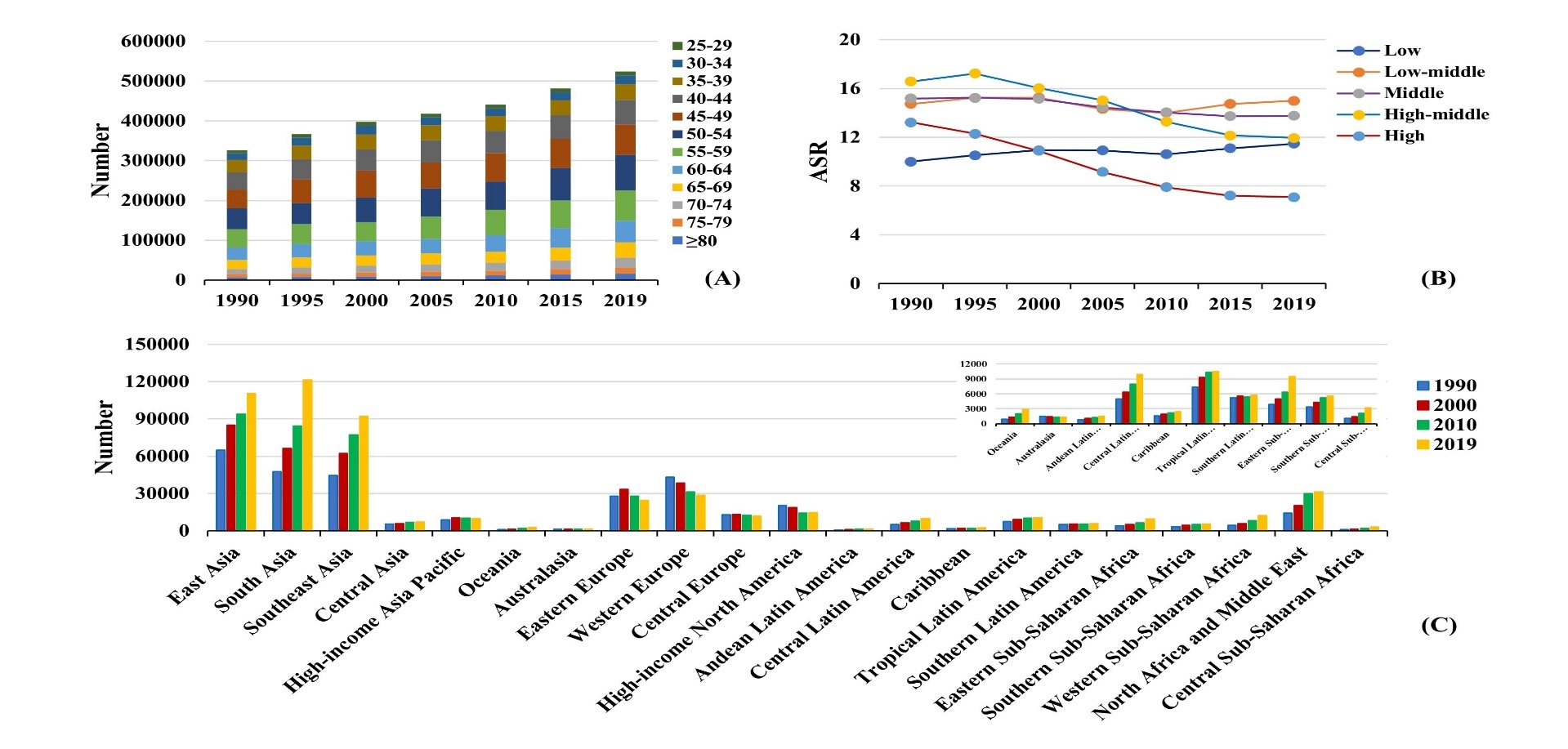
**

**Supplementary figure 2**. The distribution of ASR, percentage, and EAPC of YLLs caused by breast cancer attributable to secondhand smoke at the national level. (A) was the ASR of YLLs in 2019; (B) was the percentage changes in number of YLLs between 2000 and 2019; (C) was the EAPCs of YLLs, respectively. Countries/territories with an extreme value were annotated. ASR, age-standardized rate; EAPC, estimated annual percentage change; YLLs, years of life lost.


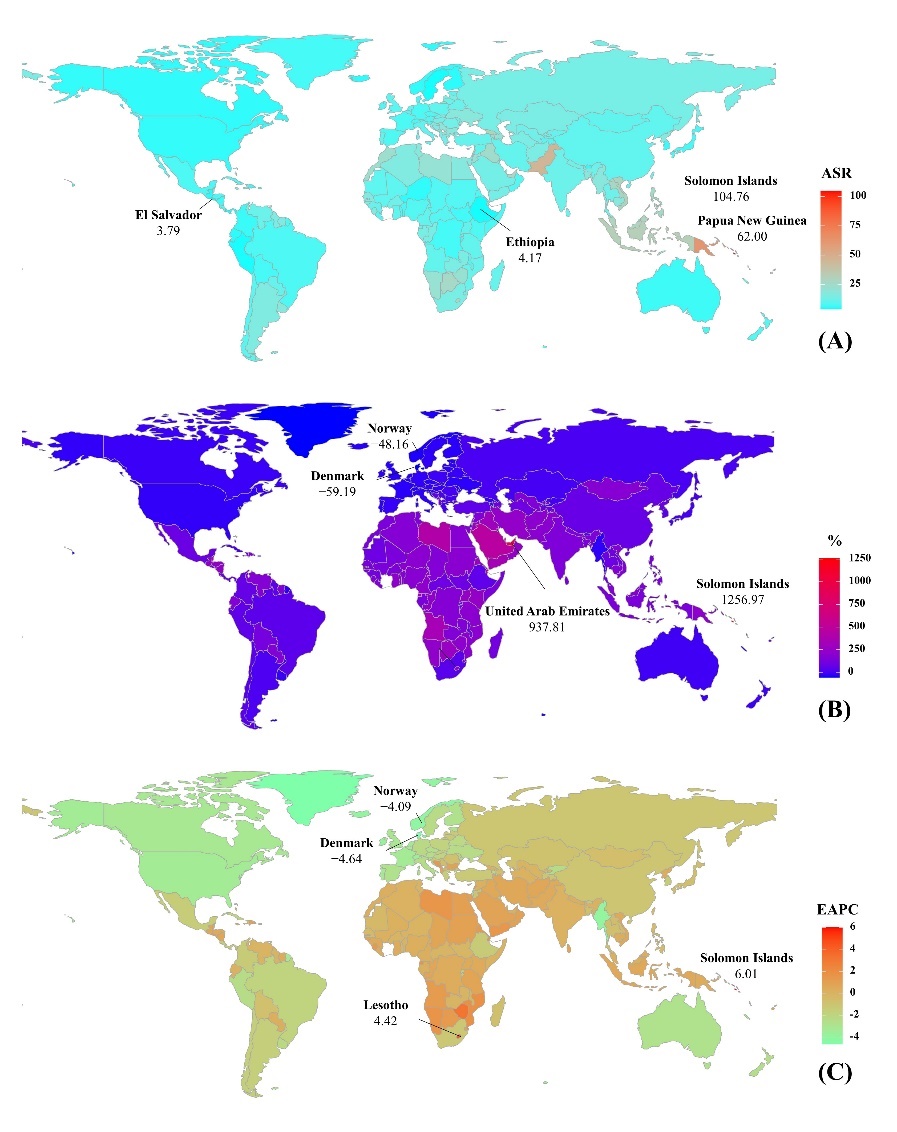


**Supplementary figure 3**. The distribution of YLDs caused by breast cancer attributable to secondhand smoke from 1990 to 2019. (A) was the number of YLDs in age groups; (B) was the ASR of YLDs in SDI areas; (C) was the number of YLDs in geographical regions. ASR, age-standardized rate; SDI, sociodemographic index; YLDs, years lived with disability.

**
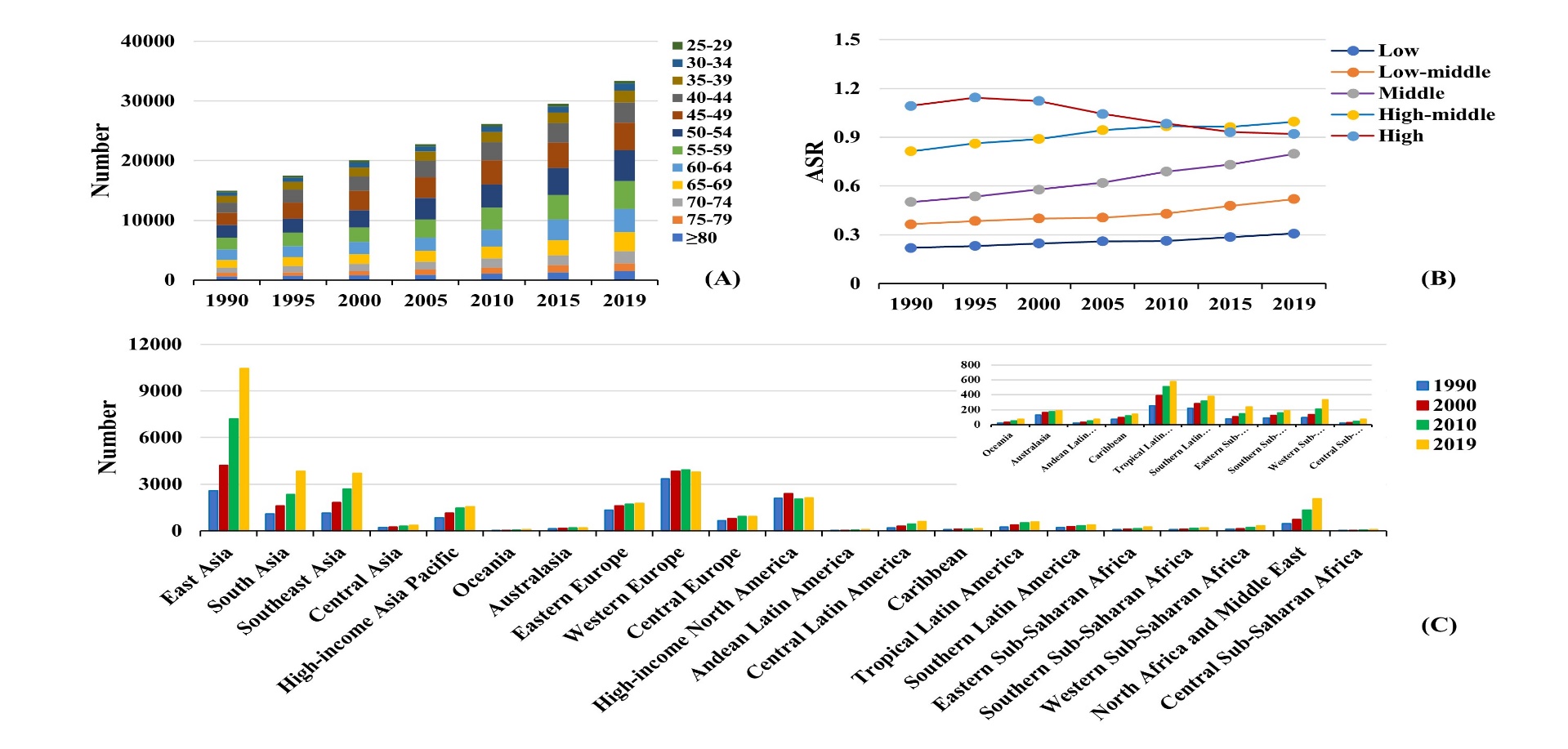
**

**Supplementary figure 4**. The distribution of ASR, percentage, and EAPC of YLDs caused by breast cancer attributable to secondhand smoke at the national level. (A) was the ASR of YLDs in 2019; (B) was the percentage changes in number of YLDs between 2000 and 2019; (C) was the EAPCs of YLDs, respectively. Countries/territories with an extreme value were annotated. ASR, age-standardized rate; EAPC, estimated annual percentage change; YLDs, years lived with disability.

**
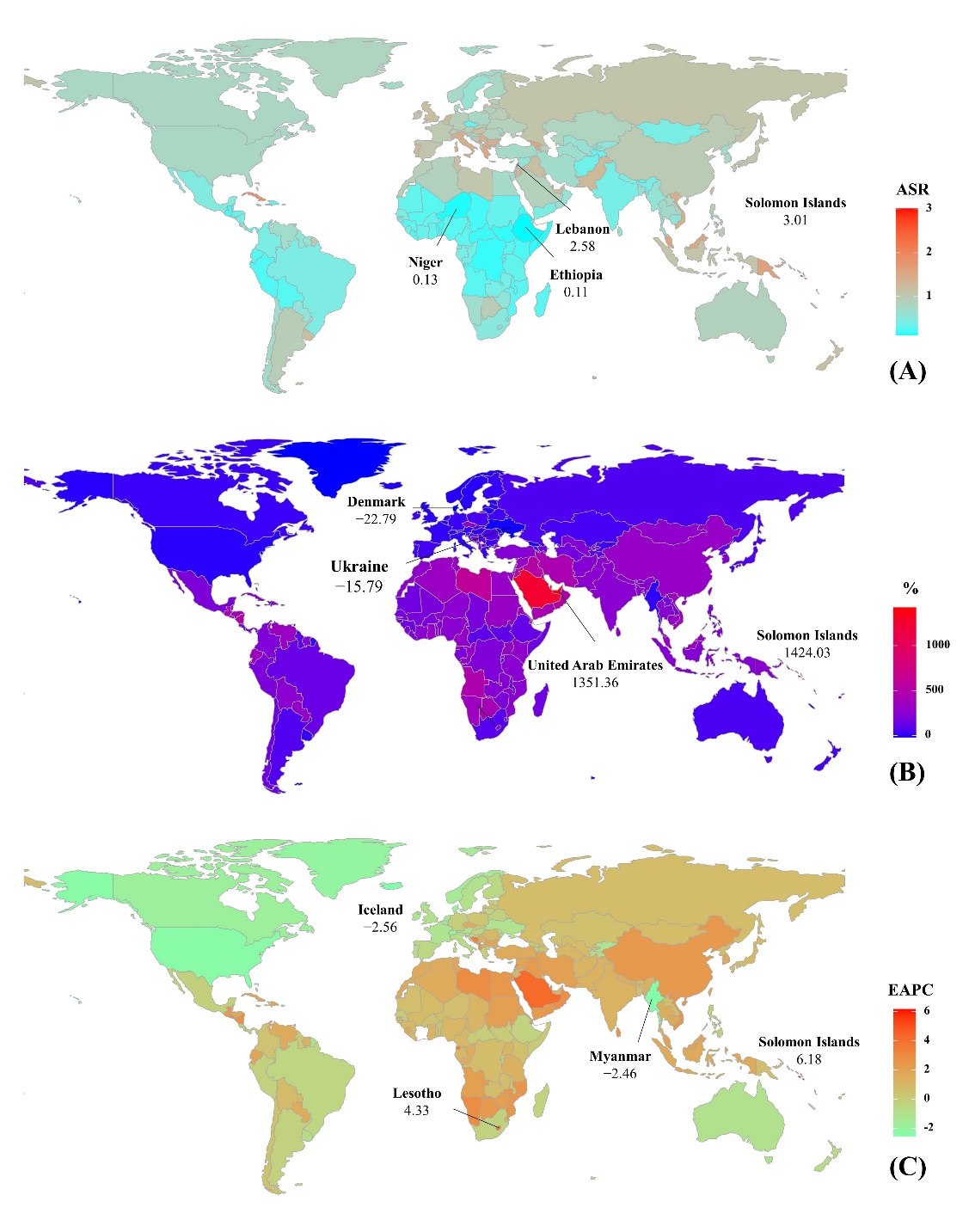
**
