# Supplementary material for: Global Trends in Death, Years of Life Lost, and Years Lived With Disability Caused by Breast Cancer Attributable to Secondhand Smoke From 1990 to 2019
Source: Front Oncol. 2022 Mar 29;12:853038. doi: 10.3389/fonc.2022.853038 (PMC9001985; doi:10.3389/fonc.2022.853038)
Supplement: Supplementary file 2 [file Table_1.docx]

**Supplementary table 1**. the characteristics and percentages of death, YLLs, and YLDs caused by breast cancer attributable to secondhand smoke in age groups, 1990-2019.

| **Age Groups** | **Death** | | **YLLs** | | **YLDs** | |
| --- | --- | --- | --- | --- | --- | --- |
|  | Number  ×10^2^(95% UI) | Percentage (%) | Number  ×10^2^(95% UI) | Percentage (%) | Number  ×10^2^(95% UI) | Percentage (%) |
| **25 to 29** | 1.55(0.37-2.7) | 39.97 | 95.45(22.83-166.32) | 39.95 | 4.76(1.03-8.79) | 91.52 |
| **30 to 34** | 3.95(0.93-6.78) | 33.18 | 223.66(52.61-383.55) | 33.15 | 11.48(2.49-21.07) | 95.08 |
| **35 to 39** | 7.71(1.84-13.41) | 26.21 | 398.17(94.83-692.51) | 26.17 | 19.93(4.29-36.48) | 77.59 |
| **40 to 44** | 13.1(3.15-22.46) | 42.79 | 612.35(147.01-1050.03) | 42.78 | 34.26(7.52-63.39) | 95.59 |
| **45 to 49** | 18.23(4.26-31.33) | 60.74 | 762.95(178.46-1311.49) | 60.69 | 45.98(10.06-84.03) | 127.31 |
| **50 to 54** | 23.97(5.6-41.18) | 69.24 | 889.8(207.85-1528.72) | 69.22 | 51.56(11.67-94.75) | 140.73 |
| **55 to 59** | 23.67(5.66-40.7) | 68.33 | 767.75(183.59-1319.72) | 68.3 | 47.15(10.54-85.28) | 136.13 |
| **60 to 64** | 19.47(4.67-33.35) | 66.9 | 541.59(130.06-927.84) | 66.9 | 38.96(8.71-70.62) | 127.72 |
| **65 to 69** | 16.64(3.93-29.24) | 78.91 | 388.34(91.65-682.24) | 78.74 | 31.38(7.23-58.3) | 134.65 |
| **70 to 74** | 13.19(3.2-22.6) | 95.55 | 251.68(61.11-431.18) | 95.44 | 20.64(4.73-37.74) | 145.12 |
| **75 to 79** | 9.8(2.24-16.99) | 79.47 | 147.63(33.69-255.83) | 78.98 | 12.59(2.87-22.82) | 110 |
| **>80** | 17.04(4.11-29.9) | 145.85 | 163.21(39.11-286.47) | 135.52 | 15.34(3.58-27.62) | 155.79 |

YLLs, years of life lost; YLDs, years lived with disability.

**Supplementary table 2**. the characteristics and changes of death caused by breast cancer attributable to secondhand smoke at national level from 1990 to 2019.

|  | **1990** | | **2019** | | **1990−2019** | |
| --- | --- | --- | --- | --- | --- | --- |
| **Characteristics** | Number  ×10^2^ (95% UI) | ASR/100,000  (95% UI) | Number  ×10^2^ (95% UI) | ASR/100,000  (95% UI) | Percentage (%) | EAPC  (95%CI) |
| Afghanistan | 0.13(0.03-0.24) | 0.36(0.08-0.65) | 0.37(0.09-0.69) | 0.46(0.11-0.86) | 181.1 | 0.82(0.79-0.85) |
| Albania | 0.04(0.01-0.06) | 0.33(0.08-0.57) | 0.08(0.02-0.15) | 0.39(0.1-0.74) | 110.9 | 0.77(0.56-0.98) |
| Algeria | 0.32(0.07-0.56) | 0.5(0.12-0.86) | 0.93(0.23-1.69) | 0.52(0.13-0.95) | 188.62 | 0.25(0.14-0.35) |
| American Samoa | 0 | 0.82(0.18-1.43) | 0 | 1.07(0.26-1.94) | 175.92 | 0.92(0.8-1.04) |
| Andorra | 0 | 0.39(0.08-0.74) | 0 | 0.27(0.06-0.51) | 81.6 | −1.49(−1.68-−1.3) |
| Angola | 0.05(0.01-0.11) | 0.22(0.05-0.43) | 0.25(0.05-0.46) | 0.33(0.07-0.61) | 356.07 | 1.45(1.38-1.52) |
| Antigua and Barbuda | 0 | 0.28(0.06-0.49) | 0 | 0.34(0.08-0.61) | 135.01 | 0.48(0.32-0.65) |
| Argentina | 1.42(0.34-2.5) | 0.82(0.2-1.44) | 1.76(0.4-3.09) | 0.59(0.13-1.04) | 23.84 | −1.44(−1.59-−1.3) |
| Armenia | 0.19(0.05-0.33) | 1.2(0.3-2.04) | 0.18(0.04-0.33) | 0.82(0.18-1.44) | −3.44 | −2.01(−2.35-−1.68) |
| Australia | 0.39(0.09-0.68) | 0.41(0.1-0.71) | 0.39(0.09-0.69) | 0.21(0.05-0.36) | −0.22 | −2.63(−2.8-−2.45) |
| Austria | 0.26(0.06-0.45) | 0.45(0.11-0.78) | 0.23(0.06-0.4) | 0.26(0.07-0.45) | −11.31 | −2.06(−2.14-−1.99) |
| Azerbaijan | 0.2(0.05-0.35) | 0.66(0.16-1.16) | 0.35(0.09-0.63) | 0.62(0.15-1.08) | 78.69 | −0.36(−0.53-−0.19) |
| Bahamas | 0.01(0-0.01) | 0.57(0.13-1.01) | 0.01(0-0.02) | 0.49(0.11-0.89) | 105.68 | −0.34(−0.42-−0.26) |
| Bahrain | 0.01(0-0.01) | 0.66(0.13-1.15) | 0.02(0-0.04) | 0.52(0.12-0.92) | 276.23 | −1.05(−1.24-−0.85) |
| Bangladesh | 1.46(0.32-2.74) | 0.58(0.12-1.07) | 3.76(0.86-6.84) | 0.55(0.13-1) | 157.93 | −0.58(−0.74-−0.43) |
| Barbados | 0.01(0-0.01) | 0.38(0.08-0.68) | 0.01(0-0.02) | 0.36(0.09-0.67) | 64.88 | 0(−0.1-0.1) |
| Belarus | 0.37(0.09-0.63) | 0.51(0.12-0.87) | 0.32(0.07-0.58) | 0.36(0.08-0.67) | −13.06 | −1.97(−2.26-−1.67) |
| Belgium | 0.47(0.11-0.83) | 0.63(0.15-1.09) | 0.32(0.08-0.56) | 0.29(0.07-0.5) | −32.29 | −3.03(−3.17-−2.89) |
| Belize | 0 | 0.18(0.04-0.31) | 0(0-0.01) | 0.21(0.05-0.37) | 297.89 | 0.66(0.37-0.95) |
| Benin | 0.03(0.01-0.05) | 0.22(0.05-0.41) | 0.07(0.02-0.13) | 0.22(0.05-0.41) | 171.81 | −0.11(−0.21-−0.01) |
| Bermuda | 0 | 0.54(0.13-0.95) | 0 | 0.26(0.06-0.49) | −5.15 | −3.04(−3.27-−2.8) |
| Bhutan | 0(0-0.01) | 0.2(0.04-0.39) | 0(0-0.01) | 0.17(0.04-0.33) | 68.51 | −0.84(−1.04-−0.64) |
| Bolivia | 0.05(0.01-0.09) | 0.25(0.05-0.45) | 0.11(0.02-0.22) | 0.23(0.05-0.44) | 134.21 | −0.56(−0.71-−0.42) |
| Bosnia and Herzegovina | 0.08(0.02-0.14) | 0.34(0.08-0.6) | 0.18(0.04-0.33) | 0.57(0.14-1.06) | 114.33 | 2.08(1.82-2.34) |
| Botswana | 0.02(0.01-0.04) | 0.65(0.15-1.18) | 0.08(0.02-0.15) | 0.93(0.21-1.8) | 263.58 | 1.29(1.07-1.5) |
| Brazil | 2.25(0.53-3.89) | 0.45(0.11-0.78) | 3.45(0.79-6.04) | 0.26(0.06-0.46) | 52.97 | −1.95(−2.12-−1.78) |
| Brunei Darussalam | 0(0-0.01) | 0.47(0.1-0.87) | 0.01(0-0.02) | 0.4(0.08-0.72) | 151.56 | 0(−0.41-0.41) |
| Bulgaria | 0.35(0.09-0.61) | 0.56(0.14-0.97) | 0.4(0.1-0.74) | 0.58(0.15-1.06) | 15.56 | 1.13(0.73-1.53) |
| Burkina Faso | 0.09(0.02-0.18) | 0.39(0.09-0.72) | 0.23(0.05-0.4) | 0.4(0.09-0.72) | 139.73 | −0.03(−0.17-0.11) |
| Burundi | 0.03(0.01-0.06) | 0.23(0.05-0.42) | 0.05(0.01-0.1) | 0.19(0.05-0.37) | 59.67 | −1.05(−1.27-−0.83) |
| Cabo Verde | 0(0-0.01) | 0.24(0.06-0.43) | 0.01(0-0.01) | 0.24(0.05-0.44) | 107.15 | 0.08(−0.25-0.41) |
| Cambodia | 0.13(0.03-0.24) | 0.42(0.1-0.77) | 0.38(0.09-0.68) | 0.51(0.12-0.91) | 188.77 | 0.41(0.31-0.51) |
| Cameroon | 0.08(0.02-0.14) | 0.29(0.06-0.54) | 0.24(0.05-0.46) | 0.32(0.07-0.62) | 209.22 | 0.2(0.11-0.3) |
| Canada | 0.62(0.14-1.1) | 0.38(0.09-0.67) | 0.59(0.13-1.03) | 0.18(0.04-0.32) | −5.41 | −3(−3.21-−2.79) |
| Central African Republic | 0.02(0-0.04) | 0.28(0.06-0.53) | 0.04(0.01-0.09) | 0.28(0.06-0.59) | 90.56 | −0.07(−0.14-0) |
| Chad | 0.03(0.01-0.06) | 0.21(0.05-0.4) | 0.08(0.02-0.16) | 0.27(0.07-0.5) | 151.21 | 0.89(0.82-0.95) |
| Chile | 0.29(0.07-0.5) | 0.55(0.14-0.93) | 0.46(0.11-0.8) | 0.35(0.08-0.61) | 57.11 | −1.52(−1.6-−1.43) |
| China | 18.16(4.3-32.03) | 0.4(0.09-0.69) | 35.39(8.61-62.95) | 0.34(0.08-0.61) | 94.91 | −0.71(−0.78-−0.64) |
| Colombia | 0.3(0.07-0.52) | 0.3(0.07-0.52) | 0.56(0.13-1.06) | 0.2(0.05-0.37) | 88.31 | −1.55(−1.66-−1.43) |
| Comoros | 0(0-0.01) | 0.38(0.07-0.76) | 0.01(0-0.02) | 0.47(0.1-0.87) | 183.06 | 0.49(0.3-0.69) |
| Congo | 0.02(0-0.04) | 0.34(0.07-0.67) | 0.07(0.01-0.14) | 0.43(0.09-0.88) | 217.63 | 0.86(0.69-1.04) |
| Cook Islands | 0 | 1.7(0.37-3.14) | 0 | 1.44(0.34-2.61) | 63.29 | −0.47(−0.67-−0.27) |
| Costa Rica | 0.04(0.01-0.06) | 0.38(0.09-0.66) | 0.08(0.02-0.14) | 0.27(0.07-0.49) | 111.94 | −1.31(−1.49-−1.13) |
| Croatia | 0.28(0.07-0.49) | 0.8(0.2-1.39) | 0.26(0.06-0.48) | 0.54(0.13-1) | −9.14 | −0.83(−1.07-−0.58) |
| Cuba | 0.24(0.06-0.42) | 0.46(0.11-0.81) | 0.29(0.07-0.52) | 0.3(0.07-0.53) | 23.24 | −1.66(−1.75-−1.56) |
| Cyprus | 0.02(0.01-0.04) | 0.61(0.14-1.08) | 0.05(0.01-0.08) | 0.48(0.12-0.85) | 87.83 | −0.82(−0.98-−0.66) |
| Czechia | 0.38(0.09-0.67) | 0.52(0.12-0.92) | 0.29(0.07-0.52) | 0.28(0.07-0.5) | −24.69 | −2.35(−2.47-−2.23) |
| Côte d'Ivoire | 0.06(0.01-0.12) | 0.28(0.06-0.51) | 0.22(0.05-0.41) | 0.36(0.08-0.68) | 245.64 | 1.1(0.97-1.22) |
| Democratic People's Republic of Korea | 0.49(0.11-0.91) | 0.46(0.11-0.86) | 0.91(0.2-1.69) | 0.51(0.11-0.95) | 87.81 | 0.27(0.19-0.36) |
| Democratic Republic of the Congo | 0.18(0.04-0.35) | 0.19(0.04-0.36) | 0.5(0.11-0.97) | 0.22(0.05-0.43) | 174.23 | 0.32(0.14-0.51) |
| Denmark | 0.27(0.06-0.48) | 0.72(0.17-1.27) | 0.13(0.03-0.23) | 0.24(0.06-0.43) | −52.03 | −4.26(−4.42-−4.09) |
| Djibouti | 0(0-0.01) | 0.45(0.1-0.83) | 0.02(0-0.04) | 0.72(0.15-1.3) | 507.32 | 1.93(1.75-2.11) |
| Dominica | 0 | 0.39(0.1-0.7) | 0 | 0.39(0.09-0.71) | 19.92 | 0.08(0.01-0.16) |
| Dominican Republic | 0.03(0.01-0.06) | 0.16(0.04-0.29) | 0.1(0.02-0.19) | 0.19(0.04-0.37) | 185.18 | 0.97(0.77-1.16) |
| Ecuador | 0.05(0.01-0.08) | 0.16(0.04-0.28) | 0.13(0.03-0.24) | 0.16(0.04-0.3) | 185.44 | 0.18(0-0.37) |
| Egypt | 0.63(0.16-1.1) | 0.37(0.09-0.63) | 1.8(0.38-3.56) | 0.51(0.11-1) | 184.23 | 1.21(0.68-1.75) |
| El Salvador | 0.01(0-0.02) | 0.08(0.02-0.15) | 0.04(0.01-0.08) | 0.12(0.03-0.23) | 182.4 | 1.22(1.1-1.34) |
| Equatorial Guinea | 0(0-0.01) | 0.25(0.06-0.49) | 0.01(0-0.03) | 0.43(0.09-0.91) | 324.15 | 2.17(2.04-2.29) |
| Eritrea | 0.01(0-0.02) | 0.16(0.03-0.33) | 0.04(0.01-0.09) | 0.25(0.05-0.47) | 282.39 | 1.49(1.35-1.62) |
| Estonia | 0.06(0.01-0.1) | 0.52(0.13-0.89) | 0.04(0.01-0.07) | 0.31(0.07-0.56) | −33.83 | −2.34(−2.62-−2.06) |
| Eswatini | 0.01(0-0.01) | 0.32(0.07-0.6) | 0.01(0-0.03) | 0.36(0.08-0.7) | 120.91 | 0.74(0.31-1.17) |
| Ethiopia | 0.2(0.04-0.39) | 0.17(0.03-0.32) | 0.33(0.08-0.6) | 0.13(0.03-0.24) | 62.57 | −1.11(−1.36-−0.86) |
| Fiji | 0.02(0.01-0.04) | 1.11(0.25-1.99) | 0.05(0.01-0.09) | 1.23(0.29-2.27) | 107.75 | 0.68(0.43-0.93) |
| Finland | 0.11(0.03-0.2) | 0.32(0.07-0.56) | 0.09(0.02-0.15) | 0.17(0.04-0.29) | −24.1 | −2.51(−2.6-−2.41) |
| France | 2.03(0.49-3.5) | 0.53(0.13-0.9) | 1.46(0.36-2.59) | 0.24(0.06-0.42) | −28.05 | −3.17(−3.41-−2.92) |
| Gabon | 0.01(0-0.02) | 0.4(0.08-0.73) | 0.03(0.01-0.05) | 0.46(0.09-0.89) | 127.67 | 0.27(0.02-0.52) |
| Gambia | 0(0-0.01) | 0.22(0.05-0.4) | 0.02(0-0.03) | 0.33(0.07-0.64) | 343.78 | 1.24(1.01-1.47) |
| Georgia | 0.35(0.08-0.59) | 0.99(0.23-1.69) | 0.29(0.07-0.51) | 0.93(0.21-1.66) | −16.54 | 0.35(0-0.7) |
| Germany | 2.53(0.58-4.33) | 0.41(0.1-0.71) | 1.98(0.47-3.49) | 0.25(0.06-0.43) | −21.87 | −2.02(−2.12-−1.91) |
| Ghana | 0.1(0.02-0.18) | 0.25(0.06-0.47) | 0.32(0.07-0.61) | 0.3(0.07-0.57) | 224.83 | 0.44(0.34-0.54) |
| Greece | 0.46(0.11-0.81) | 0.62(0.15-1.07) | 0.52(0.12-0.92) | 0.45(0.1-0.8) | 11.32 | −1.23(−1.36-−1.09) |
| Greenland | 0 | 0.78(0.19-1.37) | 0 | 0.39(0.09-0.71) | −13.47 | −3.15(−3.44-−2.86) |
| Grenada | 0 | 0.39(0.09-0.7) | 0 | 0.43(0.1-0.76) | 74.27 | 0.56(0.24-0.88) |
| Guam | 0 | 0.65(0.15-1.17) | 0.01(0-0.01) | 0.58(0.15-1.05) | 129.53 | −0.61(−0.91-−0.32) |
| Guatemala | 0.03(0.01-0.05) | 0.13(0.03-0.23) | 0.1(0.02-0.19) | 0.16(0.04-0.29) | 261.96 | 0.72(0.59-0.86) |
| Guinea | 0.05(0.01-0.1) | 0.31(0.07-0.56) | 0.11(0.02-0.21) | 0.35(0.08-0.67) | 101.73 | 0.53(0.48-0.58) |
| Guinea-Bissau | 0.01(0-0.02) | 0.33(0.07-0.62) | 0.02(0-0.04) | 0.4(0.09-0.82) | 135 | 0.82(0.78-0.87) |
| Guyana | 0.01(0-0.02) | 0.51(0.11-0.89) | 0.02(0-0.03) | 0.45(0.11-0.84) | 47.26 | −0.35(−0.53-−0.16) |
| Haiti | 0.07(0.02-0.13) | 0.36(0.09-0.69) | 0.14(0.03-0.26) | 0.31(0.07-0.59) | 94.54 | −0.52(−0.63-−0.41) |
| Honduras | 0.02(0.01-0.04) | 0.2(0.04-0.37) | 0.09(0.02-0.17) | 0.27(0.06-0.5) | 270.19 | 0.97(0.77-1.16) |
| Hungary | 0.59(0.14-1.01) | 0.74(0.17-1.28) | 0.51(0.13-0.92) | 0.49(0.12-0.88) | −12.46 | −1.75(−1.97-−1.52) |
| Iceland | 0.01(0-0.01) | 0.49(0.11-0.87) | 0(0-0.01) | 0.17(0.04-0.31) | −34.12 | −3.94(−4.14-−3.73) |
| India | 8.72(2.07-15.55) | 0.36(0.09-0.65) | 23.05(5.55-42.42) | 0.38(0.09-0.7) | 164.45 | −0.09(−0.31-0.14) |
| Indonesia | 4.8(1.12-8.54) | 0.74(0.17-1.3) | 11.82(2.82-21.1) | 0.89(0.21-1.58) | 146.53 | 0.66(0.52-0.79) |
| Iran  (Islamic Republic of) | 0.41(0.1-0.73) | 0.29(0.07-0.53) | 1.39(0.33-2.43) | 0.34(0.08-0.59) | 238.75 | 0.61(0.44-0.78) |
| Iraq | 0.26(0.06-0.5) | 0.59(0.14-1.11) | 1.04(0.26-1.98) | 0.74(0.18-1.38) | 294.37 | 0.68(0.61-0.75) |
| Ireland | 0.13(0.03-0.23) | 0.68(0.16-1.17) | 0.1(0.02-0.18) | 0.28(0.07-0.49) | −22.22 | −3.42(−3.54-−3.3) |
| Israel | 0.14(0.03-0.25) | 0.6(0.14-1.05) | 0.18(0.04-0.31) | 0.31(0.08-0.55) | 25.28 | −2.8(−2.99-−2.61) |
| Italy | 2.48(0.57-4.31) | 0.58(0.13-1.01) | 1.94(0.45-3.4) | 0.31(0.07-0.54) | −21.69 | −2.29(−2.33-−2.24) |
| Jamaica | 0.03(0.01-0.06) | 0.39(0.09-0.67) | 0.08(0.02-0.15) | 0.5(0.12-0.94) | 126.5 | 0.64(0.29-0.99) |
| Japan | 2.14(0.51-3.68) | 0.24(0.06-0.42) | 2.79(0.66-4.87) | 0.22(0.05-0.37) | 30.45 | −0.44(−0.55-−0.33) |
| Jordan | 0.07(0.02-0.12) | 0.82(0.19-1.45) | 0.25(0.06-0.46) | 0.69(0.15-1.23) | 284.79 | −0.85(−1.18-−0.52) |
| Kazakhstan | 0.49(0.12-0.85) | 0.63(0.15-1.09) | 0.5(0.12-0.87) | 0.47(0.11-0.83) | 1.9 | −0.54(−0.85-−0.22) |
| Kenya | 0.1(0.02-0.21) | 0.21(0.05-0.44) | 0.35(0.08-0.68) | 0.26(0.06-0.49) | 244 | 0.6(0.56-0.64) |
| Kiribati | 0(0-0.01) | 1.23(0.29-2.21) | 0.01(0-0.01) | 1.44(0.34-2.73) | 120.98 | 0.39(0.25-0.54) |
| Kuwait | 0.02(0-0.03) | 0.59(0.14-1.03) | 0.06(0.01-0.11) | 0.41(0.09-0.74) | 225.8 | −0.8(−1.29-−0.31) |
| Kyrgyzstan | 0.11(0.03-0.19) | 0.61(0.14-1.07) | 0.1(0.02-0.18) | 0.37(0.09-0.64) | −5.66 | −2.02(−2.35-−1.68) |
| Lao People's Democratic Republic | 0.11(0.02-0.22) | 0.91(0.19-1.78) | 0.22(0.05-0.45) | 0.86(0.19-1.7) | 98.22 | −0.44(−0.51-−0.36) |
| Latvia | 0.12(0.03-0.2) | 0.59(0.15-1.01) | 0.1(0.02-0.18) | 0.48(0.11-0.89) | −16.58 | −0.85(−1.09-−0.6) |
| Lebanon | 0.09(0.02-0.17) | 0.74(0.16-1.36) | 0.31(0.07-0.56) | 1.07(0.23-1.95) | 233.16 | 1.69(1.4-1.98) |
| Lesotho | 0.02(0-0.04) | 0.35(0.08-0.67) | 0.06(0.01-0.13) | 0.84(0.19-1.65) | 227.13 | 4.32(3.79-4.85) |
| Liberia | 0.01(0-0.03) | 0.25(0.06-0.46) | 0.03(0.01-0.07) | 0.28(0.06-0.55) | 141.64 | 0.39(0.2-0.58) |
| Libya | 0.04(0.01-0.07) | 0.4(0.1-0.74) | 0.2(0.04-0.36) | 0.64(0.14-1.19) | 402.68 | 1.67(1.47-1.87) |
| Lithuania | 0.12(0.03-0.21) | 0.49(0.12-0.85) | 0.1(0.02-0.18) | 0.36(0.08-0.64) | −17.28 | −1.09(−1.27-−0.91) |
| Luxembourg | 0.02(0-0.03) | 0.64(0.15-1.12) | 0.01(0-0.03) | 0.29(0.07-0.51) | −17.23 | −2.7(−2.8-−2.6) |
| Madagascar | 0.12(0.03-0.21) | 0.39(0.09-0.7) | 0.25(0.05-0.49) | 0.34(0.08-0.67) | 112.93 | −0.7(−0.84-−0.56) |
| Malawi | 0.06(0.01-0.11) | 0.24(0.06-0.46) | 0.12(0.03-0.21) | 0.26(0.06-0.47) | 98.13 | 0.13(0-0.26) |
| Malaysia | 0.47(0.11-0.82) | 0.87(0.21-1.53) | 1.35(0.34-2.48) | 0.97(0.25-1.77) | 187.8 | 0.27(0.11-0.43) |
| Maldives | 0(0-0.01) | 0.72(0.13-1.44) | 0.01(0-0.01) | 0.54(0.13-0.95) | 138.91 | −1.56(−1.82-−1.29) |
| Mali | 0.06(0.01-0.11) | 0.26(0.06-0.46) | 0.15(0.03-0.28) | 0.31(0.07-0.57) | 141.56 | 0.34(0.21-0.48) |
| Malta | 0.02(0-0.03) | 0.66(0.15-1.16) | 0.01(0-0.02) | 0.29(0.07-0.51) | −24.58 | −2.88(−2.98-−2.78) |
| Marshall Islands | 0 | 0.89(0.2-1.58) | 0(0-0.01) | 1.35(0.28-2.69) | 230.24 | 1.22(0.94-1.5) |
| Mauritania | 0.02(0-0.04) | 0.36(0.08-0.69) | 0.04(0.01-0.07) | 0.34(0.07-0.62) | 91.25 | −0.23(−0.28-−0.18) |
| Mauritius | 0.02(0-0.04) | 0.48(0.11-0.83) | 0.07(0.02-0.14) | 0.81(0.2-1.46) | 270.97 | 1.93(1.78-2.08) |
| Mexico | 0.83(0.21-1.43) | 0.34(0.08-0.59) | 1.6(0.35-2.85) | 0.24(0.05-0.43) | 91.63 | −1.58(−1.8-−1.36) |
| Micronesia  (Federated States of) | 0 | 1.1(0.25-1.97) | 0.01(0-0.01) | 1.53(0.3-3.28) | 124.09 | 1.13(1.04-1.21) |
| Monaco | 0 | 0.55(0.12-1) | 0 | 0.46(0.1-0.84) | 9.13 | −0.45(−0.58-−0.33) |
| Mongolia | 0.02(0-0.03) | 0.3(0.07-0.55) | 0.05(0.01-0.09) | 0.32(0.07-0.57) | 174.71 | −0.39(−0.78-0) |
| Montenegro | 0.02(0-0.03) | 0.49(0.11-0.88) | 0.03(0.01-0.06) | 0.68(0.16-1.23) | 103.22 | 1.44(1.07-1.82) |
| Morocco | 0.48(0.12-0.86) | 0.59(0.15-1.05) | 1.2(0.27-2.25) | 0.66(0.15-1.23) | 148.42 | 0.3(0.27-0.33) |
| Mozambique | 0.08(0.02-0.16) | 0.23(0.05-0.43) | 0.25(0.05-0.49) | 0.37(0.07-0.69) | 199.97 | 1.9(1.66-2.14) |
| Myanmar | 1.98(0.43-3.94) | 1.39(0.31-2.73) | 1.52(0.35-2.81) | 0.55(0.13-1.01) | −23.07 | −3.52(−3.82-−3.22) |
| Namibia | 0.02(0-0.03) | 0.44(0.11-0.8) | 0.06(0.01-0.11) | 0.68(0.15-1.32) | 233.25 | 1.82(1.7-1.95) |
| Nauru | 0 | 1.27(0.27-2.45) | 0 | 1.61(0.34-3.05) | 60.81 | 0.84(0.56-1.12) |
| Nepal | 0.2(0.04-0.38) | 0.36(0.08-0.7) | 0.52(0.11-0.98) | 0.42(0.09-0.78) | 162.96 | 0.41(0.3-0.51) |
| Netherlands | 0.64(0.15-1.11) | 0.64(0.15-1.12) | 0.48(0.12-0.85) | 0.31(0.08-0.54) | −25.2 | −3.05(−3.21-−2.89) |
| New Zealand | 0.09(0.02-0.16) | 0.49(0.12-0.85) | 0.09(0.02-0.16) | 0.25(0.06-0.45) | −5.92 | −2.54(−2.68-−2.41) |
| Nicaragua | 0.01(0-0.03) | 0.16(0.04-0.27) | 0.06(0.01-0.1) | 0.23(0.05-0.41) | 292.13 | 1.21(0.97-1.44) |
| Niger | 0.03(0.01-0.05) | 0.16(0.04-0.3) | 0.08(0.02-0.16) | 0.17(0.04-0.34) | 208.59 | 0.12(0-0.24) |
| Nigeria | 0.57(0.13-1.07) | 0.26(0.06-0.49) | 1.53(0.32-2.92) | 0.28(0.06-0.52) | 170.63 | 0.34(0.18-0.49) |
| Niue | 0 | 1.01(0.23-1.89) | 0 | 1.16(0.25-2.22) | 5.79 | 0.31(0.2-0.43) |
| North Macedonia | 0.07(0.02-0.12) | 0.68(0.16-1.18) | 0.12(0.03-0.22) | 0.76(0.18-1.39) | 76.19 | 0.45(0.24-0.66) |
| Northern Mariana Islands | 0 | 1.11(0.25-2.05) | 0 | 0.9(0.22-1.62) | 117.61 | −0.74(−0.88-−0.61) |
| Norway | 0.15(0.04-0.25) | 0.48(0.12-0.82) | 0.08(0.02-0.14) | 0.18(0.04-0.32) | −44.67 | −3.87(−4.08-−3.66) |
| Oman | 0.01(0-0.01) | 0.23(0.05-0.45) | 0.02(0.01-0.05) | 0.3(0.07-0.55) | 229.56 | 1.21(0.93-1.48) |
| Pakistan | 3.28(0.76-6.27) | 1.15(0.27-2.23) | 9.18(2.15-16.75) | 1.5(0.35-2.74) | 180.33 | 0.57(0.32-0.82) |
| Palau | 0 | 1.26(0.26-2.31) | 0 | 1.3(0.32-2.34) | 111.96 | 0.08(0.04-0.11) |
| Palestine | 0.04(0.01-0.08) | 0.77(0.19-1.45) | 0.13(0.03-0.24) | 0.95(0.22-1.67) | 238.76 | 0.76(0.54-0.97) |
| Panama | 0.02(0-0.03) | 0.21(0.05-0.36) | 0.03(0.01-0.06) | 0.14(0.03-0.26) | 83.56 | −1.37(−1.6-−1.14) |
| Papua New Guinea | 0.18(0.04-0.33) | 1.63(0.37-2.94) | 0.56(0.14-1.04) | 1.82(0.45-3.37) | 203.66 | 0.36(0.29-0.44) |
| Paraguay | 0.04(0.01-0.07) | 0.33(0.08-0.57) | 0.11(0.03-0.21) | 0.37(0.09-0.7) | 179.94 | 0.38(0.19-0.57) |
| Peru | 0.14(0.03-0.26) | 0.21(0.05-0.39) | 0.24(0.06-0.45) | 0.14(0.03-0.26) | 66.89 | −2.35(−2.67-−2.03) |
| Philippines | 1.56(0.38-2.71) | 0.94(0.22-1.61) | 3.65(0.83-6.61) | 0.81(0.18-1.45) | 133.31 | −0.96(−1.23-−0.68) |
| Poland | 1.35(0.32-2.33) | 0.56(0.13-0.97) | 1.42(0.31-2.59) | 0.38(0.08-0.7) | 5.49 | −1.56(−1.67-−1.45) |
| Portugal | 0.47(0.11-0.81) | 0.68(0.16-1.17) | 0.37(0.09-0.66) | 0.32(0.08-0.57) | −20.8 | −2.99(−3.15-−2.84) |
| Puerto Rico | 0.05(0.01-0.09) | 0.27(0.06-0.48) | 0.06(0.02-0.12) | 0.2(0.05-0.38) | 23.77 | −1.39(−1.56-−1.23) |
| Qatar | 0(0-0.01) | 0.77(0.19-1.43) | 0.03(0.01-0.05) | 0.89(0.2-1.64) | 511.02 | 0.8(0.56-1.05) |
| Republic of Korea | 0.5(0.12-0.85) | 0.26(0.06-0.44) | 0.82(0.2-1.4) | 0.18(0.04-0.31) | 65.13 | −1.36(−1.44-−1.29) |
| Republic of Moldova | 0.14(0.03-0.24) | 0.55(0.13-0.94) | 0.1(0.02-0.18) | 0.32(0.07-0.57) | −28.77 | −1.49(−1.75-−1.22) |
| Romania | 0.69(0.16-1.21) | 0.47(0.11-0.82) | 0.84(0.2-1.49) | 0.45(0.11-0.79) | 22.14 | −0.42(−0.63-−0.22) |
| Russian Federation | 4.94(1.23-8.49) | 0.47(0.12-0.8) | 5.76(1.38-10.59) | 0.44(0.1-0.81) | 16.54 | −0.83(−1.31-−0.35) |
| Rwanda | 0.07(0.01-0.13) | 0.35(0.08-0.68) | 0.11(0.03-0.21) | 0.27(0.07-0.51) | 66.61 | −1.44(−1.77-−1.12) |
| Saint Kitts and Nevis | 0 | 0.85(0.18-1.49) | 0 | 0.43(0.1-0.81) | 0.11 | −2.22(−2.4-−2.04) |
| Saint Lucia | 0 | 0.4(0.09-0.71) | 0(0-0.01) | 0.27(0.06-0.49) | 65.69 | −1.6(−1.92-−1.28) |
| Saint Vincent and the Grenadines | 0 | 0.47(0.1-0.85) | 0(0-0.01) | 0.46(0.11-0.83) | 71.79 | −0.2(−0.48-0.09) |
| Samoa | 0(0-0.01) | 0.88(0.2-1.64) | 0.01(0-0.02) | 0.95(0.21-2.1) | 82.26 | 0.13(0.08-0.18) |
| San Marino | 0 | 0.35(0.08-0.63) | 0 | 0.28(0.06-0.58) | 46.37 | −0.48(−0.65-−0.31) |
| Sao Tome and Principe | 0 | 0.11(0.02-0.22) | 0 | 0.17(0.04-0.36) | 185.07 | 1.09(0.91-1.27) |
| Saudi Arabia | 0.08(0.02-0.17) | 0.28(0.06-0.54) | 0.46(0.1-0.84) | 0.42(0.1-0.77) | 444.34 | 0.96(0.66-1.27) |
| Senegal | 0.1(0.02-0.17) | 0.55(0.13-1) | 0.22(0.05-0.41) | 0.54(0.11-0.99) | 132.31 | −0.32(−0.5-−0.13) |
| Serbia | 0.41(0.09-0.71) | 0.68(0.16-1.18) | 0.56(0.14-1.02) | 0.71(0.17-1.28) | 38.7 | −0.11(−0.36-0.15) |
| Seychelles | 0 | 0.52(0.11-0.91) | 0(0-0.01) | 0.78(0.2-1.4) | 203.08 | 1.34(1.11-1.57) |
| Sierra Leone | 0.04(0.01-0.07) | 0.39(0.09-0.73) | 0.1(0.02-0.2) | 0.52(0.12-1.01) | 171.04 | 1.17(1.07-1.28) |
| Singapore | 0.04(0.01-0.06) | 0.25(0.06-0.44) | 0.07(0.02-0.12) | 0.17(0.04-0.29) | 91.48 | −1.32(−1.49-−1.15) |
| Slovakia | 0.19(0.05-0.32) | 0.59(0.14-1.01) | 0.2(0.05-0.37) | 0.4(0.1-0.74) | 6.35 | −1.33(−1.52-−1.15) |
| Slovenia | 0.08(0.02-0.15) | 0.58(0.13-1.07) | 0.07(0.01-0.12) | 0.3(0.07-0.54) | −15.26 | −2.33(−2.43-−2.23) |
| Solomon Islands | 0(0-0.01) | 0.66(0.14-1.28) | 0.06(0.01-0.11) | 2.96(0.68-5.38) | 1162.86 | 5.65(5.02-6.29) |
| Somalia | 0.04(0.01-0.08) | 0.24(0.05-0.45) | 0.1(0.02-0.21) | 0.23(0.05-0.48) | 146.1 | 0.1(0.02-0.18) |
| South Africa | 0.86(0.21-1.51) | 0.69(0.17-1.21) | 1.34(0.33-2.34) | 0.52(0.13-0.91) | 55.78 | −0.94(−1.11-−0.77) |
| South Sudan | 0.03(0.01-0.05) | 0.23(0.05-0.43) | 0.05(0.01-0.1) | 0.23(0.05-0.45) | 86.29 | 0.04(−0.09-0.17) |
| Spain | 1.35(0.33-2.32) | 0.52(0.13-0.89) | 1.17(0.27-2.05) | 0.26(0.06-0.45) | −13.4 | −2.57(−2.7-−2.44) |
| Sri Lanka | 0.19(0.04-0.33) | 0.31(0.07-0.55) | 0.5(0.13-0.97) | 0.37(0.09-0.7) | 169.87 | 1(0.87-1.13) |
| Sudan | 0.1(0.02-0.19) | 0.19(0.05-0.38) | 0.29(0.07-0.57) | 0.26(0.07-0.5) | 187.55 | 1.1(0.99-1.21) |
| Suriname | 0.01(0-0.01) | 0.41(0.1-0.73) | 0.02(0-0.03) | 0.49(0.12-0.9) | 170.52 | 0.47(0.26-0.68) |
| Sweden | 0.15(0.04-0.27) | 0.25(0.06-0.43) | 0.12(0.03-0.2) | 0.14(0.03-0.24) | −24.43 | −2.05(−2.1-−2) |
| Switzerland | 0.32(0.08-0.55) | 0.62(0.15-1.07) | 0.2(0.05-0.35) | 0.24(0.06-0.43) | −37.26 | −3.41(−3.64-−3.18) |
| Syrian Arab Republic | 0.08(0.02-0.16) | 0.27(0.06-0.52) | 0.23(0.04-0.43) | 0.33(0.06-0.63) | 176.65 | 0.33(0.05-0.61) |
| Taiwan  (Province of China) | 0.31(0.08-0.53) | 0.37(0.09-0.63) | 0.7(0.15-1.26) | 0.35(0.08-0.65) | 125.79 | −0.33(−0.55-−0.1) |
| Tajikistan | 0.05(0.01-0.1) | 0.34(0.08-0.61) | 0.09(0.02-0.16) | 0.27(0.06-0.49) | 60.94 | −0.76(−1.03-−0.5) |
| Thailand | 0.93(0.22-1.68) | 0.43(0.1-0.77) | 1.98(0.48-3.73) | 0.36(0.09-0.68) | 112.58 | −0.77(−1.1-−0.43) |
| Timor-Leste | 0.01(0-0.02) | 0.58(0.13-1.09) | 0.03(0.01-0.05) | 0.63(0.14-1.19) | 146.99 | 0.39(0.09-0.69) |
| Togo | 0.02(0-0.04) | 0.25(0.06-0.44) | 0.06(0.01-0.12) | 0.24(0.05-0.46) | 199.93 | −0.3(−0.46-−0.13) |
| Tokelau | 0 | 1.1(0.24-2.16) | 0 | 1.31(0.31-2.5) | 13.61 | 0.58(0.5-0.66) |
| Tonga | 0(0-0.01) | 1.18(0.26-2.12) | 0.01(0-0.01) | 1.22(0.29-2.24) | 45.64 | −0.12(−0.23-−0.01) |
| Trinidad and Tobago | 0.03(0.01-0.05) | 0.6(0.13-1.04) | 0.05(0.01-0.09) | 0.53(0.13-0.99) | 87.6 | −0.33(−0.5-−0.17) |
| Tunisia | 0.14(0.03-0.25) | 0.54(0.12-0.95) | 0.36(0.08-0.7) | 0.54(0.12-1.04) | 157.44 | −0.18(−0.32-−0.04) |
| Turkey | 1(0.22-1.87) | 0.5(0.11-0.92) | 1.74(0.4-3.15) | 0.37(0.08-0.67) | 73.79 | −1.02(−1.24-−0.81) |
| Turkmenistan | 0.05(0.01-0.09) | 0.46(0.11-0.8) | 0.1(0.02-0.19) | 0.43(0.09-0.77) | 90.42 | −0.11(−0.59-0.37) |
| Tuvalu | 0 | 1.19(0.27-2.16) | 0 | 1.37(0.29-2.7) | 50.27 | 0.39(0.31-0.47) |
| Uganda | 0.09(0.02-0.16) | 0.22(0.05-0.43) | 0.29(0.07-0.55) | 0.3(0.07-0.57) | 239.49 | 0.54(0.22-0.86) |
| Ukraine | 3.17(0.76-5.49) | 0.8(0.19-1.38) | 2.18(0.52-4.03) | 0.54(0.13-0.99) | −31.26 | −2.2(−2.54-−1.86) |
| United Arab Emirates | 0.02(0-0.03) | 0.83(0.2-1.6) | 0.15(0.03-0.28) | 0.85(0.2-1.57) | 872.82 | 0.17(−0.5-0.85) |
| United Kingdom | 2.87(0.62-4.96) | 0.67(0.15-1.16) | 1.84(0.44-3.2) | 0.31(0.07-0.55) | −35.85 | −2.93(−3.08-−2.78) |
| United Republic of Tanzania | 0.17(0.04-0.31) | 0.29(0.07-0.52) | 0.51(0.11-0.92) | 0.36(0.08-0.64) | 196.76 | 0.74(0.64-0.83) |
| United States of America | 6.04(1.46-10.56) | 0.39(0.09-0.68) | 4.85(1.18-8.42) | 0.18(0.04-0.31) | −19.69 | −3.1(−3.3-−2.9) |
| United States Virgin Islands | 0 | 0.4(0.1-0.73) | 0(0-0.01) | 0.33(0.08-0.62) | 51.07 | −0.83(−0.9-−0.75) |
| Uruguay | 0.23(0.05-0.39) | 1.1(0.26-1.88) | 0.2(0.05-0.35) | 0.67(0.16-1.15) | −10.67 | −2.01(−2.12-−1.91) |
| Uzbekistan | 0.22(0.05-0.39) | 0.34(0.08-0.59) | 0.54(0.13-0.96) | 0.4(0.1-0.71) | 143.94 | 0.18(−0.1-0.47) |
| Vanuatu | 0 | 0.59(0.14-1.12) | 0.01(0-0.02) | 0.88(0.19-1.76) | 299.49 | 1.05(0.75-1.35) |
| Venezuela | 0.21(0.05-0.35) | 0.37(0.09-0.64) | 0.61(0.14-1.16) | 0.38(0.09-0.74) | 194.28 | 0.03(−0.1-0.15) |
| Viet Nam | 1.89(0.43-3.49) | 0.81(0.19-1.5) | 5.05(1.09-9.38) | 0.91(0.2-1.68) | 166.66 | 0.58(0.48-0.67) |
| Yemen | 0.08(0.02-0.16) | 0.27(0.06-0.57) | 0.33(0.07-0.64) | 0.4(0.09-0.75) | 324.89 | 1.53(1.45-1.61) |
| Zambia | 0.07(0.02-0.14) | 0.44(0.09-0.8) | 0.19(0.04-0.36) | 0.46(0.1-0.86) | 161.19 | 0(−0.11-0.12) |
| Zimbabwe | 0.1(0.02-0.17) | 0.43(0.1-0.75) | 0.3(0.06-0.57) | 0.66(0.14-1.24) | 203.39 | 2.96(2.32-3.61) |

EAPC: estimated annual percentage change; ASR, age-standardized rate; CI, confidence interval; UI: uncertainty interval. ASR, age-standardized rate.

**Supplementary table 3**. the characteristics and changes of YLLs caused by breast cancer attributable to secondhand smoke at the national level from 1990 to 2019.

| **Characteristics** | **1990** | | **2019** | | **1990−2019** | |
| --- | --- | --- | --- | --- | --- | --- |
|  | Number  ×10^2^ (95% UI) | ASR/100,000  (95% UI) | Number  ×10^2^ (95% UI) | ASR/100,000  (95% UI) | Percentage (%) | EAPC  (95%CI) |
| Afghanistan | 4.61(1.07-8.52) | 11.74(2.73-21.62) | 13.79(3.53-25.79) | 14.32(3.56-26.55) | 198.8 | 0.62(0.58-0.66) |
| Albania | 1.27(0.32-2.16) | 10.56(2.66-17.97) | 2.23(0.56-4.24) | 11.86(2.96-22.64) | 76.05 | 0.64(0.41-0.87) |
| Algeria | 11.42(2.57-19.95) | 15.66(3.57-27.33) | 31.19(7.69-57.43) | 15.53(3.89-28.47) | 173.19 | 0.01(−0.1-0.12) |
| American Samoa | 0.03(0.01-0.06) | 23.22(5.07-41.14) | 0.08(0.02-0.15) | 31.12(7.67-56.82) | 154.01 | 1.02(0.9-1.14) |
| Andorra | 0.03(0.01-0.06) | 11.89(2.58-22.68) | 0.05(0.01-0.1) | 8.25(1.89-15.48) | 62.96 | −1.53(−1.71-−1.36) |
| Angola | 2.05(0.47-3.94) | 7.31(1.66-14.21) | 9.03(1.96-16.87) | 10.35(2.25-19.28) | 340.54 | 1.22(1.14-1.3) |
| Antigua and Barbuda | 0.02(0.01-0.04) | 8.66(1.96-15.3) | 0.05(0.01-0.1) | 9.39(2.26-17.14) | 136.25 | 0.17(−0.03-0.36) |
| Argentina | 38.2(9.17-66.65) | 22.27(5.36-38.76) | 42.42(9.68-74.59) | 15.44(3.54-27.19) | 11.05 | −1.61(−1.74-−1.47) |
| Armenia | 6.36(1.58-10.81) | 38.9(9.73-66.36) | 5.23(1.18-9.26) | 24.24(5.54-43.1) | −17.73 | −2.36(−2.71-−2.01) |
| Australia | 12.33(2.98-21.27) | 13.29(3.23-22.87) | 10.83(2.64-18.97) | 6.33(1.53-11.11) | −12.15 | −2.83(−3-−2.66) |
| Austria | 7.4(1.75-12.97) | 14.47(3.44-25.25) | 5.47(1.35-9.51) | 7.55(1.88-13.21) | −26.13 | −2.51(−2.61-−2.41) |
| Azerbaijan | 6.74(1.61-11.83) | 22.01(5.36-38.82) | 11.88(3.01-20.96) | 19.23(4.91-33.77) | 76.34 | −0.72(−0.88-−0.57) |
| Bahamas | 0.19(0.04-0.34) | 18.41(4.21-33.28) | 0.36(0.08-0.67) | 15.37(3.35-28.4) | 92.03 | −0.5(−0.57-−0.43) |
| Bahrain | 0.21(0.04-0.36) | 18.76(3.8-33.17) | 0.76(0.17-1.36) | 13.56(2.99-24.29) | 269.48 | −1.45(−1.64-−1.25) |
| Bangladesh | 53.69(12.11-100.73) | 19.38(4.24-36.34) | 124.86(28.46-227.25) | 17.09(3.91-31.25) | 132.55 | −0.84(−1-−0.68) |
| Barbados | 0.16(0.03-0.28) | 11.73(2.6-21.14) | 0.25(0.06-0.46) | 10.72(2.62-19.83) | 59.69 | −0.21(−0.29-−0.13) |
| Belarus | 11.71(2.9-20.1) | 17.25(4.23-29.67) | 9.06(2.01-16.57) | 11.31(2.51-20.78) | −22.62 | −2.31(−2.6-−2.01) |
| Belgium | 12.87(2.94-22.52) | 19.23(4.46-33.37) | 7.44(1.77-12.89) | 8.22(1.95-14.23) | −42.14 | −3.32(−3.46-−3.18) |
| Belize | 0.03(0.01-0.05) | 5.43(1.25-9.65) | 0.11(0.03-0.21) | 6.56(1.55-11.78) | 332.14 | 0.61(0.32-0.91) |
| Benin | 0.87(0.19-1.57) | 6.93(1.52-12.74) | 2.42(0.54-4.57) | 7.03(1.56-13.18) | 179.25 | −0.15(−0.25-−0.04) |
| Bermuda | 0.05(0.01-0.09) | 15.27(3.57-26.65) | 0.04(0.01-0.07) | 7.09(1.64-13.17) | −24.82 | −3.22(−3.47-−2.97) |
| Bhutan | 0.11(0.02-0.22) | 6.69(1.38-13.4) | 0.16(0.04-0.32) | 5.17(1.17-10.02) | 48.78 | −1.28(−1.51-−1.06) |
| Bolivia | 1.65(0.32-3.05) | 8.02(1.55-14.76) | 3.57(0.81-6.97) | 7.01(1.59-13.76) | 116.61 | −0.76(−0.9-−0.62) |
| Bosnia and Herzegovina | 2.66(0.63-4.62) | 10.79(2.59-18.66) | 4.35(1.08-8.11) | 15.23(3.79-28.37) | 63.12 | 1.37(1.17-1.57) |
| Botswana | 0.67(0.16-1.26) | 18.3(4.4-33.94) | 2.48(0.55-4.91) | 25.81(5.8-51.14) | 269.62 | 1.32(1.12-1.52) |
| Brazil | 72.35(16.88-125.18) | 13.27(3.11-22.93) | 101.67(22.95-178.24) | 7.74(1.75-13.58) | 40.52 | −1.92(−2.08-−1.76) |
| Brunei Darussalam | 0.13(0.03-0.24) | 16.88(3.52-31.02) | 0.31(0.07-0.56) | 13.43(2.85-24.48) | 129.66 | −0.21(−0.67-0.25) |
| Bulgaria | 10.62(2.6-18.56) | 17.8(4.35-31.03) | 10.21(2.56-18.76) | 17.08(4.25-31.34) | −3.86 | 0.79(0.42-1.17) |
| Burkina Faso | 3.16(0.74-5.99) | 11.44(2.67-21.22) | 7.74(1.73-14.24) | 11.77(2.66-21.21) | 144.99 | −0.07(−0.23-0.1) |
| Burundi | 1.23(0.23-2.33) | 7.78(1.48-14.72) | 1.98(0.46-3.94) | 6.29(1.48-12.59) | 61.4 | −1.2(−1.42-−0.97) |
| Cabo Verde | 0.09(0.02-0.16) | 7.7(1.76-13.95) | 0.17(0.04-0.31) | 6.89(1.51-12.25) | 92.34 | −0.32(−0.62-−0.02) |
| Cambodia | 4.97(1.16-9.08) | 14.69(3.44-26.85) | 13.03(2.98-23.67) | 16.63(3.81-30.14) | 161.91 | 0.19(0.08-0.3) |
| Cameroon | 2.74(0.58-5.03) | 9.35(1.99-17.12) | 8.65(1.83-16.94) | 10.21(2.19-19.71) | 216.1 | 0.2(0.11-0.29) |
| Canada | 18.67(4.28-33.23) | 11.79(2.69-20.9) | 15.24(3.4-26.7) | 5.43(1.2-9.6) | −18.41 | −3.17(−3.39-−2.95) |
| Central African Republic | 0.76(0.16-1.48) | 9.14(1.9-17.75) | 1.44(0.28-3.18) | 8.85(1.73-19.37) | 90.58 | −0.16(−0.24-−0.09) |
| Chad | 1.16(0.28-2.2) | 6.78(1.64-12.81) | 3.04(0.75-5.78) | 8.27(2.06-15.68) | 162 | 0.77(0.7-0.84) |
| Chile | 8.18(2.01-14.04) | 14.33(3.51-24.6) | 11.02(2.66-19.18) | 8.89(2.16-15.53) | 34.77 | −1.63(−1.74-−1.53) |
| China | 620.48(148.99-1112.23) | 12.76(3.06-22.63) | 1052.76(253.25-1880.78) | 10.17(2.43-18.23) | 69.67 | −1.05(−1.15-−0.95) |
| Colombia | 10.07(2.4-17.53) | 9.15(2.18-15.88) | 16.87(3.91-32.13) | 6.02(1.39-11.47) | 67.58 | −1.46(−1.61-−1.31) |
| Comoros | 0.15(0.03-0.32) | 11.71(2.16-24.25) | 0.42(0.09-0.79) | 14.19(3.15-26.68) | 172.07 | 0.36(0.11-0.61) |
| Congo | 0.77(0.16-1.55) | 10.96(2.25-21.76) | 2.5(0.47-5.24) | 13.36(2.61-27.55) | 223.57 | 0.72(0.55-0.89) |
| Cook Islands | 0.03(0.01-0.06) | 50.02(10.76-92.39) | 0.05(0.01-0.08) | 39.58(9.5-72.81) | 35.42 | −0.67(−0.91-−0.43) |
| Costa Rica | 1.15(0.27-2) | 11.39(2.66-19.85) | 2.17(0.53-3.99) | 7.77(1.9-14.27) | 88.32 | −1.49(−1.69-−1.29) |
| Croatia | 7.55(1.85-13.21) | 22.17(5.47-38.9) | 5.33(1.28-9.87) | 13.31(3.19-24.95) | −29.45 | −1.37(−1.58-−1.16) |
| Cuba | 7.15(1.69-12.47) | 13.74(3.25-23.96) | 7.55(1.75-13.57) | 8.4(1.92-15.12) | 5.7 | −1.93(−2.04-−1.83) |
| Cyprus | 0.71(0.17-1.26) | 17.39(4.03-30.54) | 1.2(0.3-2.14) | 12.8(3.21-22.85) | 67.7 | −1.11(−1.29-−0.93) |
| Czechia | 10.77(2.49-18.79) | 15.81(3.66-27.71) | 6.78(1.68-12.3) | 7.63(1.92-13.82) | −37.07 | −2.67(−2.78-−2.57) |
| Côte d'Ivoire | 2.27(0.51-4.26) | 8.34(1.91-15.42) | 7.59(1.74-14.5) | 10.64(2.36-20.17) | 234.49 | 1.01(0.9-1.13) |
| Democratic People's Republic of Korea | 16.7(3.85-31.82) | 15.39(3.58-29.18) | 29.32(6.22-55.98) | 16.86(3.55-32.38) | 75.56 | 0.25(0.17-0.33) |
| Democratic Republic of the Congo | 6.54(1.51-12.61) | 5.96(1.38-11.42) | 17.71(3.8-33.65) | 6.95(1.54-13.35) | 170.86 | 0.36(0.18-0.54) |
| Denmark | 7.65(1.79-13.56) | 22.6(5.26-40.05) | 3.12(0.72-5.54) | 6.89(1.55-12.32) | −59.19 | −4.64(−4.82-−4.46) |
| Djibouti | 0.13(0.03-0.25) | 13.11(2.95-24.6) | 0.77(0.14-1.45) | 19.79(3.68-36.29) | 477.49 | 1.64(1.47-1.81) |
| Dominica | 0.04(0.01-0.07) | 12.16(2.9-21.84) | 0.05(0.01-0.09) | 11.61(2.74-21.45) | 19.23 | −0.16(−0.23-−0.09) |
| Dominican Republic | 1.17(0.28-2.1) | 5.02(1.18-8.96) | 3.06(0.64-6) | 5.93(1.24-11.66) | 161.52 | 0.89(0.68-1.1) |
| Ecuador | 1.61(0.36-2.88) | 4.95(1.11-8.84) | 4.23(0.99-7.77) | 5.06(1.19-9.31) | 163.48 | 0.05(−0.13-0.24) |
| Egypt | 23.29(5.79-40.45) | 12.34(3.07-21.57) | 64.43(13.7-127.36) | 16.43(3.49-32.47) | 176.59 | 1.05(0.52-1.58) |
| El Salvador | 0.5(0.12-0.9) | 2.83(0.67-5.03) | 1.28(0.28-2.57) | 3.79(0.84-7.63) | 153.04 | 1.06(0.94-1.17) |
| Equatorial Guinea | 0.11(0.03-0.21) | 8.16(1.95-15.66) | 0.45(0.09-0.94) | 12.26(2.49-25.52) | 302.57 | 1.69(1.55-1.82) |
| Eritrea | 0.44(0.08-0.91) | 5.32(0.99-10.9) | 1.6(0.35-3.1) | 7.74(1.72-14.93) | 264.47 | 1.29(1.19-1.4) |
| Estonia | 1.83(0.44-3.12) | 16.79(4.02-28.76) | 1.01(0.23-1.83) | 9.29(2.07-17) | −44.81 | −2.7(−3.02-−2.39) |
| Eswatini | 0.19(0.04-0.37) | 9.45(2.05-18.1) | 0.42(0.09-0.84) | 10.44(2.19-20.84) | 115.63 | 0.76(0.31-1.22) |
| Ethiopia | 7.66(1.52-15.16) | 5.66(1.18-11.14) | 11.83(2.76-21.9) | 4.17(0.98-7.65) | 54.38 | −1.45(−1.7-−1.2) |
| Fiji | 0.86(0.2-1.56) | 33.9(7.76-61.28) | 1.59(0.37-3.01) | 36.18(8.49-68.3) | 85.71 | 0.57(0.3-0.83) |
| Finland | 3.46(0.81-6.05) | 10.48(2.45-18.48) | 2.14(0.5-3.77) | 5.06(1.2-9.01) | −38.05 | −2.82(−2.93-−2.7) |
| France | 60.33(14.79-104.17) | 17.22(4.25-29.62) | 37.44(9.29-65.6) | 7.59(1.87-13.2) | −37.94 | −3.34(−3.59-−3.1) |
| Gabon | 0.39(0.08-0.72) | 12.2(2.63-22.53) | 0.91(0.19-1.8) | 13.38(2.79-26.32) | 134.63 | 0.14(−0.13-0.41) |
| Gambia | 0.13(0.03-0.25) | 6.42(1.47-12.02) | 0.57(0.12-1.13) | 9.72(2.02-19.28) | 330.09 | 1.23(0.98-1.47) |
| Georgia | 11.11(2.58-18.95) | 33.13(7.68-56.6) | 8.04(1.81-14.33) | 28.89(6.51-51.64) | −27.6 | −0.01(−0.34-0.33) |
| Germany | 76.25(17.94-131.41) | 13.75(3.31-23.79) | 51.82(12.2-91.78) | 7.64(1.78-13.5) | −32.04 | −2.28(−2.4-−2.16) |
| Ghana | 3.68(0.87-7.01) | 8.39(2-15.9) | 11.55(2.58-22.33) | 9.69(2.16-18.49) | 213.38 | 0.31(0.21-0.41) |
| Greece | 13.03(3.09-22.47) | 18.49(4.4-31.85) | 11.41(2.62-20.28) | 12.4(2.86-21.88) | −12.48 | −1.47(−1.53-−1.4) |
| Greenland | 0.05(0.01-0.09) | 25.3(6.15-44.58) | 0.04(0.01-0.08) | 12.07(2.62-22.22) | −22.2 | −3.16(−3.38-−2.94) |
| Grenada | 0.04(0.01-0.07) | 12.42(2.73-22.27) | 0.07(0.02-0.13) | 12.73(2.98-22.98) | 81.78 | 0.33(0.02-0.64) |
| Guam | 0.08(0.02-0.14) | 17(4.01-30.51) | 0.16(0.04-0.29) | 17.54(4.54-31.71) | 110.28 | −0.05(−0.24-0.14) |
| Guatemala | 1.02(0.25-1.83) | 4.29(1.08-7.7) | 3.31(0.78-6.2) | 4.79(1.12-8.86) | 225.95 | 0.52(0.36-0.68) |
| Guinea | 1.84(0.41-3.35) | 9.67(2.2-17.5) | 3.8(0.84-7.18) | 10.91(2.41-20.79) | 105.99 | 0.48(0.42-0.54) |
| Guinea-Bissau | 0.3(0.06-0.59) | 10.84(2.3-20.63) | 0.72(0.16-1.49) | 12.82(2.77-26.77) | 134.93 | 0.73(0.68-0.78) |
| Guyana | 0.37(0.08-0.66) | 15.36(3.22-27.39) | 0.54(0.13-1.02) | 14.41(3.45-26.83) | 47.19 | −0.16(−0.39-0.06) |
| Haiti | 2.55(0.53-5.07) | 12.14(2.63-23.66) | 4.93(1.07-9.9) | 9.93(2.19-19.59) | 93.12 | −0.64(−0.76-−0.52) |
| Honduras | 0.88(0.19-1.59) | 6.72(1.45-12.11) | 2.89(0.69-5.43) | 7.83(1.87-14.63) | 229.31 | 0.5(0.36-0.64) |
| Hungary | 15.9(3.71-27.32) | 21.5(5.04-37.01) | 11.61(2.88-20.79) | 13.09(3.23-23.71) | −26.96 | −2.05(−2.27-−1.83) |
| Iceland | 0.2(0.05-0.35) | 15.29(3.57-27.28) | 0.12(0.03-0.22) | 5.31(1.24-9.57) | −38.88 | −3.98(−4.2-−3.77) |
| India | 306.09(72.18-542.84) | 10.91(2.59-19.4) | 753.46(179.04-1359.1) | 11.7(2.79-21.19) | 146.16 | 0.01(−0.22-0.25) |
| Indonesia | 190.51(44.3-341.58) | 26.99(6.28-48.09) | 443.45(105.14-805.48) | 31.43(7.45-56.9) | 132.78 | 0.55(0.42-0.69) |
| Iran  (Islamic Republic of) | 14.6(3.62-25.71) | 9.15(2.24-16.23) | 47.78(11.15-83.32) | 10.61(2.48-18.56) | 227.26 | 0.57(0.4-0.73) |
| Iraq | 9.53(2.23-17.8) | 20.11(4.73-37.58) | 37.67(9.31-72.05) | 24.25(5.97-46.17) | 295.04 | 0.56(0.49-0.64) |
| Ireland | 3.85(0.91-6.65) | 21(4.96-36.12) | 2.7(0.65-4.74) | 7.88(1.9-13.73) | −29.68 | −3.77(−3.91-−3.63) |
| Israel | 4.55(1.05-7.87) | 19.63(4.53-33.92) | 4.95(1.21-8.73) | 9.61(2.34-16.98) | 8.83 | −2.97(−3.13-−2.8) |
| Italy | 73.36(16.84-127.3) | 18.72(4.33-32.76) | 49.17(11.64-85.75) | 9.47(2.28-16.48) | −32.97 | −2.48(−2.53-−2.43) |
| Jamaica | 1.02(0.24-1.76) | 11.94(2.79-20.56) | 2.45(0.57-4.58) | 16.12(3.78-30.13) | 140.15 | 0.88(0.51-1.25) |
| Japan | 70.19(16.32-120.72) | 8.25(1.9-14.19) | 70.52(16.96-121.08) | 6.92(1.69-11.89) | 0.46 | −0.7(−0.84-−0.56) |
| Jordan | 2.35(0.55-4.12) | 26.2(6.16-46.17) | 8.63(1.94-15.67) | 20.76(4.63-37.65) | 267.28 | −1.12(−1.48-−0.75) |
| Kazakhstan | 15.92(3.93-27.66) | 20.36(5.03-35.47) | 15.5(3.71-27.17) | 14.45(3.46-25.34) | −2.63 | −0.9(−1.21-−0.58) |
| Kenya | 3.58(0.78-7.69) | 6.68(1.44-14.21) | 12.31(2.74-24.13) | 7.92(1.79-15.41) | 244.01 | 0.5(0.45-0.56) |
| Kiribati | 0.1(0.02-0.18) | 39.84(9.58-72.2) | 0.22(0.05-0.42) | 44.41(11.03-85.02) | 114.71 | 0.22(0.09-0.34) |
| Kuwait | 0.67(0.16-1.15) | 17.83(4.21-30.79) | 2.08(0.48-3.76) | 11.33(2.59-20.37) | 211.55 | −1.3(−1.81-−0.78) |
| Kyrgyzstan | 3.45(0.82-6.02) | 19.95(4.75-35.14) | 3.19(0.74-5.61) | 10.77(2.5-18.94) | −7.36 | −2.45(−2.79-−2.11) |
| Lao People's Democratic Republic | 3.97(0.78-8.01) | 30.11(6-60.32) | 7.7(1.7-15.46) | 26.87(5.82-54.18) | 93.75 | −0.59(−0.66-−0.52) |
| Latvia | 3.6(0.9-6.21) | 19.04(4.74-32.95) | 2.45(0.56-4.57) | 14.14(3.14-26.17) | −31.9 | −1.29(−1.56-−1.02) |
| Lebanon | 3.12(0.7-5.68) | 23.8(5.35-43.24) | 9.11(1.98-16.77) | 31.73(6.86-58.63) | 192.28 | 1.36(1.12-1.59) |
| Lesotho | 0.62(0.14-1.2) | 10.61(2.44-20.62) | 2.08(0.46-4.21) | 25.27(5.62-50.88) | 236.97 | 4.42(3.85-4.99) |
| Liberia | 0.46(0.11-0.82) | 7.81(1.82-14.17) | 1.18(0.24-2.39) | 8.26(1.7-16.85) | 157.58 | 0.26(0.06-0.45) |
| Libya | 1.37(0.33-2.51) | 13.32(3.18-24.43) | 7.03(1.49-13.01) | 20.68(4.41-38.16) | 412.76 | 1.57(1.36-1.77) |
| Lithuania | 3.91(0.94-6.7) | 16.5(3.96-28.4) | 2.68(0.63-4.82) | 11.09(2.59-19.93) | −31.52 | −1.38(−1.58-−1.19) |
| Luxembourg | 0.49(0.11-0.87) | 19.16(4.44-33.56) | 0.37(0.09-0.65) | 8.15(1.97-14.43) | −25.06 | −2.92(−3.01-−2.84) |
| Madagascar | 4.29(0.97-8.13) | 12.91(2.92-24.23) | 9.16(2.01-18.62) | 11.01(2.44-21.95) | 113.35 | −0.8(−0.96-−0.65) |
| Malawi | 2.14(0.48-4.02) | 8.16(1.84-15.3) | 4.02(0.98-7.55) | 7.94(1.95-14.76) | 87.61 | −0.2(−0.35-−0.05) |
| Malaysia | 16.28(4.03-28.26) | 27.53(6.76-47.99) | 42.88(10.83-78.78) | 28.86(7.27-52.93) | 163.38 | 0.14(−0.06-0.33) |
| Maldives | 0.12(0.02-0.25) | 22.9(4.1-46.86) | 0.25(0.06-0.44) | 14.83(3.6-25.99) | 105.44 | −2.09(−2.36-−1.81) |
| Mali | 2.23(0.53-3.95) | 8.61(2.04-15.16) | 5.28(1.17-9.77) | 9.74(2.15-17.83) | 137.12 | 0.15(0-0.3) |
| Malta | 0.46(0.1-0.8) | 20.22(4.58-35.73) | 0.3(0.07-0.52) | 8.83(2.13-15.58) | −34.99 | −2.82(−2.93-−2.71) |
| Marshall Islands | 0.03(0.01-0.05) | 28.22(6.15-50.02) | 0.1(0.02-0.21) | 42.72(8.5-87.19) | 236.98 | 1.22(0.93-1.5) |
| Mauritania | 0.64(0.14-1.25) | 10.98(2.43-21.29) | 1.22(0.24-2.28) | 9.71(1.94-18.08) | 89.14 | −0.4(−0.46-−0.33) |
| Mauritius | 0.67(0.16-1.17) | 14.76(3.51-25.63) | 2.23(0.53-4.02) | 24.81(5.93-45.04) | 232.86 | 1.89(1.73-2.06) |
| Mexico | 28.17(6.91-48.36) | 10.42(2.57-17.88) | 51.56(11.14-92.28) | 7.61(1.65-13.63) | 83.04 | −1.5(−1.71-−1.29) |
| Micronesia  (Federated States of) | 0.09(0.02-0.17) | 34.56(7.99-61.94) | 0.21(0.04-0.46) | 46.55(9-103.16) | 117.63 | 1.03(0.94-1.12) |
| Monaco | 0.04(0.01-0.08) | 17.1(3.88-31.32) | 0.05(0.01-0.08) | 13.91(3.16-25.74) | 5.08 | −0.56(−0.67-−0.45) |
| Mongolia | 0.62(0.14-1.13) | 9.93(2.29-18.16) | 1.73(0.38-3.24) | 9.96(2.17-18.52) | 179.99 | −0.64(−1.01-−0.26) |
| Montenegro | 0.48(0.11-0.86) | 13.78(3.23-24.74) | 0.87(0.2-1.57) | 18.18(4.2-33.05) | 82.12 | 1.22(0.8-1.65) |
| Morocco | 18.18(4.63-32.19) | 21.13(5.36-37.54) | 43.16(9.9-82.09) | 22.68(5.2-43.25) | 137.36 | 0.18(0.14-0.22) |
| Mozambique | 3.01(0.62-5.69) | 7.3(1.49-13.73) | 8.86(1.74-17.32) | 11.05(2.16-21.29) | 194.43 | 1.81(1.52-2.1) |
| Myanmar | 75.45(15.64-152.49) | 50.03(10.64-100.6) | 50.21(11.52-93.96) | 17.11(3.95-32) | −33.45 | −4.02(−4.35-−3.69) |
| Namibia | 0.59(0.14-1.1) | 14.01(3.31-25.94) | 1.93(0.41-3.82) | 20.94(4.49-41) | 225.76 | 1.69(1.55-1.82) |
| Nauru | 0.01(0-0.02) | 38.94(8.26-76.03) | 0.02(0-0.03) | 48.89(9.94-94.59) | 62.32 | 0.83(0.57-1.1) |
| Nepal | 7.06(1.58-13.44) | 11.95(2.68-22.67) | 17.11(3.75-32.04) | 12.89(2.81-24.17) | 142.45 | 0.18(0.07-0.3) |
| Netherlands | 18.41(4.17-32.06) | 20.1(4.52-35.06) | 12.07(2.98-21.23) | 9.02(2.22-15.8) | −34.47 | −3.29(−3.46-−3.11) |
| New Zealand | 3(0.73-5.17) | 16.55(4.06-28.61) | 2.6(0.58-4.62) | 8.24(1.82-14.69) | −13.06 | −2.6(−2.73-−2.46) |
| Nicaragua | 0.53(0.12-0.92) | 5.2(1.24-9.09) | 1.77(0.41-3.19) | 6.37(1.44-11.49) | 232.66 | 0.69(0.56-0.81) |
| Niger | 0.96(0.23-1.79) | 5.1(1.18-9.5) | 2.84(0.66-5.64) | 5.35(1.24-10.39) | 194.73 | −0.09(−0.24-0.06) |
| Nigeria | 18.86(4.38-35.89) | 8.08(1.89-15.37) | 53.68(11.08-104.08) | 8.46(1.77-16.19) | 184.69 | 0.21(0.05-0.37) |
| Niue | 0(0-0.01) | 30.51(6.78-57.51) | 0(0-0.01) | 33.97(7.52-65.56) | 7.65 | 0.21(0.1-0.32) |
| North Macedonia | 2.24(0.52-3.89) | 21.38(4.95-37.04) | 3.24(0.77-5.96) | 20.48(4.86-37.39) | 44.12 | −0.23(−0.41-−0.05) |
| Northern Mariana Islands | 0.04(0.01-0.08) | 32.32(7.29-59.5) | 0.07(0.02-0.13) | 25.73(6.54-47.22) | 74.92 | −0.78(−0.93-−0.64) |
| Norway | 3.95(0.96-6.74) | 14.93(3.62-25.61) | 2.05(0.48-3.59) | 5.35(1.27-9.36) | −48.16 | −4.09(−4.31-−3.87) |
| Oman | 0.25(0.06-0.49) | 6.74(1.53-13.44) | 0.81(0.18-1.49) | 8.03(1.77-14.84) | 229.12 | 0.84(0.51-1.17) |
| Pakistan | 110.71(25.58-208.81) | 35.69(8.27-67.93) | 318.88(73.33-584.92) | 45.26(10.59-82.49) | 188.03 | 0.45(0.18-0.72) |
| Palau | 0.02(0-0.04) | 37.2(7.55-68.88) | 0.04(0.01-0.08) | 37.66(9.13-69.26) | 102.37 | 0.05(0.01-0.08) |
| Palestine | 1.36(0.31-2.59) | 24.93(5.83-47.51) | 4.59(1.07-8.23) | 29.04(6.73-51.64) | 237.61 | 0.56(0.36-0.77) |
| Panama | 0.55(0.13-0.96) | 6.57(1.52-11.43) | 0.92(0.23-1.73) | 4.32(1.08-8.1) | 66.48 | −1.44(−1.7-−1.19) |
| Papua New Guinea | 6.96(1.61-12.59) | 55.99(12.63-100.69) | 21.41(5.46-39.97) | 62(15.68-115.15) | 207.73 | 0.34(0.26-0.41) |
| Paraguay | 1.28(0.31-2.22) | 9.98(2.43-17.32) | 3.37(0.82-6.4) | 10.95(2.65-20.72) | 162.51 | 0.19(0.01-0.37) |
| Peru | 4.91(1.17-8.94) | 6.82(1.63-12.32) | 7.45(1.8-14.19) | 4.27(1.03-8.13) | 51.84 | −2.48(−2.8-−2.16) |
| Philippines | 53(12.58-91.24) | 27.54(6.58-47.59) | 123.3(28.01-224.74) | 25.52(5.8-46.44) | 132.66 | −0.59(−0.84-−0.35) |
| Poland | 38.2(9.01-66.17) | 16.62(3.94-28.83) | 32.76(6.68-60.53) | 10.14(2.01-18.79) | −14.22 | −1.89(−2-−1.78) |
| Portugal | 13.87(3.32-23.73) | 21.5(5.14-36.74) | 8.91(2.1-15.56) | 9.53(2.26-16.69) | −35.79 | −3.23(−3.39-−3.07) |
| Puerto Rico | 1.67(0.4-2.92) | 8.84(2.09-15.43) | 1.7(0.41-3.22) | 6.28(1.52-11.84) | 2.06 | −1.55(−1.74-−1.36) |
| Qatar | 0.16(0.04-0.3) | 22.79(5.62-42.05) | 0.96(0.23-1.79) | 21.26(4.88-39.21) | 497.73 | −0.08(−0.29-0.13) |
| Republic of Korea | 17.88(4.37-30.69) | 8.73(2.13-14.94) | 24.54(5.99-42.5) | 5.8(1.43-10.03) | 37.2 | −1.56(−1.62-−1.5) |
| Republic of Moldova | 4.8(1.14-8.32) | 19.1(4.56-32.92) | 2.97(0.68-5.32) | 10.21(2.34-18.27) | −38.03 | −1.87(−2.12-−1.61) |
| Romania | 21.27(5.03-37.51) | 14.88(3.53-26.22) | 20.88(5.12-37.04) | 12.85(3.14-22.89) | −1.88 | −0.82(−1.02-−0.63) |
| Russian Federation | 155.3(38.86-267.39) | 15.51(3.86-26.66) | 163.35(38.68-299.39) | 13.61(3.18-25.1) | 5.18 | −1.13(−1.61-−0.64) |
| Rwanda | 2.45(0.52-4.88) | 12.19(2.66-24.07) | 3.9(0.93-7.51) | 8.79(2.1-16.68) | 59.22 | −1.77(−2.12-−1.43) |
| Saint Kitts and Nevis | 0.04(0.01-0.08) | 26.28(5.65-46.66) | 0.04(0.01-0.08) | 11.82(2.48-22.71) | 1.42 | −2.82(−3.03-−2.6) |
| Saint Lucia | 0.06(0.01-0.11) | 12.95(2.98-22.86) | 0.1(0.02-0.18) | 8.54(1.97-15.63) | 60.01 | −1.54(−1.85-−1.22) |
| Saint Vincent and the Grenadines | 0.05(0.01-0.1) | 14.52(3.24-26.17) | 0.09(0.02-0.17) | 14.12(3.32-25.55) | 73.46 | −0.34(−0.59-−0.1) |
| Samoa | 0.13(0.03-0.25) | 27.06(6.01-51.38) | 0.24(0.05-0.53) | 29.32(6.36-64.93) | 81.98 | 0.16(0.11-0.21) |
| San Marino | 0.02(0-0.03) | 10.34(2.37-18.79) | 0.02(0-0.04) | 8.52(1.86-17.54) | 43.25 | −0.37(−0.54-−0.2) |
| Sao Tome and Principe | 0.01(0-0.03) | 3.77(0.78-7.38) | 0.04(0.01-0.09) | 5.47(1.15-11.85) | 203.18 | 0.99(0.78-1.21) |
| Saudi Arabia | 3.11(0.71-6.1) | 9.06(2.09-17.94) | 17.55(3.81-32.48) | 13.06(2.95-23.96) | 464.47 | 0.79(0.5-1.08) |
| Senegal | 3.23(0.78-5.88) | 16.5(3.98-30.12) | 7.22(1.54-13.36) | 15.42(3.24-28.62) | 123.34 | −0.44(−0.65-−0.24) |
| Serbia | 11.83(2.73-20.45) | 19.69(4.56-33.9) | 13.19(3.26-24.05) | 18.34(4.55-33.47) | 11.47 | −0.65(−0.95-−0.36) |
| Seychelles | 0.05(0.01-0.09) | 17.44(3.8-30.5) | 0.15(0.04-0.27) | 24.56(6.15-44.18) | 199.44 | 1.19(0.99-1.39) |
| Sierra Leone | 1.19(0.28-2.27) | 11.16(2.63-21.22) | 3.39(0.76-6.7) | 15.02(3.38-29.68) | 185.32 | 1.22(1.11-1.33) |
| Singapore | 1.3(0.31-2.27) | 8.54(2.02-14.93) | 2.09(0.52-3.69) | 5.2(1.28-9.2) | 61.12 | −1.67(−1.84-−1.49) |
| Slovakia | 5.48(1.36-9.37) | 17.81(4.41-30.44) | 4.79(1.18-8.86) | 10.68(2.65-19.86) | −12.61 | −1.85(−2.02-−1.67) |
| Slovenia | 2.2(0.5-4.06) | 17.1(3.89-31.47) | 1.44(0.32-2.64) | 7.87(1.69-14.44) | −34.53 | −2.78(−2.91-−2.65) |
| Solomon Islands | 0.18(0.04-0.35) | 21.36(4.46-41) | 2.46(0.58-4.56) | 104.76(24.3-191.79) | 1256.97 | 6.01(5.32-6.7) |
| Somalia | 1.54(0.3-3.06) | 7.65(1.47-14.91) | 3.64(0.78-7.54) | 7.2(1.59-14.84) | 135.83 | −0.05(−0.12-0.03) |
| South Africa | 27.83(6.6-48.01) | 20.47(4.84-35.49) | 38.53(9.32-67.82) | 13.94(3.4-24.55) | 38.42 | −1.18(−1.33-−1.02) |
| South Sudan | 0.91(0.2-1.69) | 6.97(1.56-13.03) | 1.71(0.37-3.48) | 6.64(1.51-13.24) | 89.41 | −0.21(−0.37-−0.04) |
| Spain | 40.35(10-69.1) | 17.03(4.25-28.96) | 29.96(7.01-52.71) | 7.93(1.84-13.95) | −25.76 | −2.81(−2.96-−2.66) |
| Sri Lanka | 6.48(1.57-11.43) | 9.64(2.33-17.09) | 14.66(3.63-28.58) | 10.59(2.63-20.61) | 126.34 | 0.74(0.61-0.86) |
| Sudan | 3.65(0.82-6.97) | 6.44(1.47-12.58) | 10.6(2.45-21.49) | 8.38(2.13-16.73) | 190.2 | 0.98(0.86-1.1) |
| Suriname | 0.19(0.05-0.34) | 12.61(3.02-22.61) | 0.49(0.12-0.91) | 14.8(3.57-27.15) | 159.42 | 0.36(0.15-0.57) |
| Sweden | 4.62(1.07-8.13) | 8.25(1.88-14.59) | 3.04(0.71-5.3) | 4.19(0.98-7.34) | −34.08 | −2.34(−2.4-−2.28) |
| Switzerland | 8.78(2.16-15.09) | 19.07(4.7-32.72) | 4.83(1.19-8.48) | 6.87(1.7-12.03) | −44.99 | −3.73(−3.94-−3.51) |
| Syrian Arab Republic | 3.01(0.71-5.66) | 9.2(2.18-17.42) | 7.78(1.5-14.96) | 10.38(2-19.81) | 158.43 | 0.1(−0.2-0.41) |
| Taiwan  (Province of China) | 11.07(2.78-19.03) | 12.38(3.12-21.33) | 21.49(4.67-39.25) | 11.52(2.53-21.08) | 94.18 | −0.45(−0.68-−0.22) |
| Tajikistan | 1.91(0.46-3.41) | 11.93(2.85-21.31) | 3.15(0.71-5.81) | 8.51(1.9-15.54) | 65.21 | −1.2(−1.44-−0.96) |
| Thailand | 32.8(7.7-59.13) | 13.79(3.25-24.86) | 61.54(15.12-117.47) | 11.6(2.85-22.01) | 87.64 | −0.8(−1.21-−0.38) |
| Timor-Leste | 0.4(0.09-0.75) | 18.63(4.28-35.62) | 0.87(0.19-1.67) | 19.79(4.38-37.79) | 117.65 | 0.25(−0.08-0.59) |
| Togo | 0.77(0.18-1.44) | 8.08(1.91-14.88) | 2.26(0.5-4.31) | 7.89(1.73-14.89) | 194.43 | −0.29(−0.46-−0.12) |
| Tokelau | 0 | 34.79(7.78-67.85) | 0(0-0.01) | 40.02(9.46-77.74) | 14.4 | 0.47(0.4-0.55) |
| Tonga | 0.12(0.03-0.22) | 36.72(8.16-66.3) | 0.16(0.04-0.3) | 36.76(9.28-67.69) | 33.78 | −0.24(−0.35-−0.13) |
| Trinidad and Tobago | 0.84(0.19-1.45) | 17.98(4.12-31.39) | 1.42(0.34-2.7) | 15.6(3.75-29.51) | 69.9 | −0.44(−0.62-−0.27) |
| Tunisia | 4.71(1.03-8.23) | 16.58(3.67-29.01) | 11.59(2.7-22.34) | 16.75(3.87-32.3) | 146.29 | −0.1(−0.24-0.04) |
| Turkey | 33.2(7.32-61.84) | 15.47(3.4-28.64) | 51.44(11.47-94.27) | 10.74(2.4-19.62) | 54.94 | −1.32(−1.5-−1.13) |
| Turkmenistan | 1.86(0.45-3.22) | 15.36(3.7-26.5) | 3.6(0.78-6.44) | 14.31(3.12-25.69) | 93.24 | 0.01(−0.5-0.53) |
| Tuvalu | 0.02(0-0.03) | 37.99(8.4-69.21) | 0.02(0-0.04) | 41.59(8.55-82.9) | 37.74 | 0.28(0.22-0.34) |
| Uganda | 3.14(0.7-5.95) | 7.45(1.67-14.18) | 10.81(2.36-20.57) | 10.03(2.23-18.9) | 243.69 | 0.42(0.05-0.79) |
| Ukraine | 99.09(24-171.26) | 26.73(6.62-46.43) | 64.24(15.4-119.36) | 17.39(4.16-32.04) | −35.17 | −2.41(−2.79-−2.03) |
| United Arab Emirates | 0.57(0.13-1.05) | 24.38(5.82-46.56) | 5.88(1.35-11.01) | 25.54(6.04-47.18) | 937.81 | 0.15(−0.3-0.6) |
| United Kingdom | 78.52(17.2-136.13) | 20.82(4.59-35.97) | 45.72(10.77-79.46) | 9.21(2.14-15.99) | −41.78 | −3.13(−3.29-−2.97) |
| United Republic of Tanzania | 5.72(1.33-10.34) | 8.45(1.95-15.22) | 17.06(3.66-30.64) | 10.47(2.28-18.74) | 198.12 | 0.8(0.69-0.91) |
| United States of America | 184.19(44.13-323.94) | 12.64(3.01-22.22) | 129.45(31.63-224.62) | 5.47(1.33-9.51) | −29.72 | −3.37(−3.6-−3.15) |
| United States Virgin Islands | 0.06(0.02-0.12) | 11.96(2.84-21.87) | 0.08(0.02-0.15) | 9.35(2.2-17.76) | 21.11 | −1.02(−1.11-−0.94) |
| Uruguay | 5.83(1.41-9.93) | 30.58(7.39-52.03) | 4.41(1.05-7.65) | 17.42(4.09-30.26) | −24.26 | −2.22(−2.32-−2.11) |
| Uzbekistan | 7.53(1.83-13.23) | 11.14(2.71-19.41) | 19.35(4.57-33.98) | 12.42(2.96-21.9) | 156.89 | −0.12(−0.42-0.18) |
| Vanuatu | 0.08(0.02-0.15) | 18.96(4.49-36.31) | 0.3(0.06-0.62) | 29.08(6.06-59.18) | 294.07 | 1.12(0.82-1.42) |
| Venezuela | 6.86(1.58-11.79) | 11.18(2.59-19.24) | 18.21(4.11-34.69) | 11.36(2.57-21.65) | 165.35 | −0.06(−0.2-0.08) |
| Viet Nam | 61.78(13.53-113.34) | 26.06(5.74-48.04) | 161.78(35.73-299.34) | 28(6.23-51.8) | 161.84 | 0.5(0.38-0.61) |
| Yemen | 2.8(0.62-5.75) | 8.88(1.98-18.4) | 12.2(2.61-23.43) | 13.08(2.91-24.95) | 335.03 | 1.49(1.4-1.58) |
| Zambia | 2.74(0.56-5.13) | 14.13(2.92-26.08) | 7.03(1.54-13.24) | 14.33(3.08-26.8) | 156.09 | −0.16(−0.28-−0.04) |
| Zimbabwe | 3.44(0.81-6.07) | 13.3(3.05-23.51) | 10.69(2.22-20.07) | 20.98(4.32-39.46) | 210.56 | 3.19(2.47-3.92) |

EAPC: estimated annual percentage change; ASR, age-standardized rate; CI, confidence interval; UI: uncertainty interval; YLLs, years of life lost.

**Supplementary table 4**. the characteristics and changes of YLDs caused by breast cancer attributable to secondhand smoke at the national level from 1990 to 2019.

| **Characteristics** | **1990** | | **2019** | | **1990−2019** | |
| --- | --- | --- | --- | --- | --- | --- |
|  | Number  ×10^2^ (95% UI) | ASR/100,000  (95% UI) | Number  ×10^2^ (95% UI) | ASR/100,000  (95% UI) | Percentage (%) | EAPC  (95%CI) |
| Afghanistan | 0.1(0.02-0.2) | 0.26(0.06-0.5) | 0.33(0.07-0.65) | 0.35(0.08-0.69) | 232.26 | 1.15(1.04-1.26) |
| Albania | 0.06(0.01-0.1) | 0.48(0.1-0.88) | 0.17(0.04-0.34) | 0.93(0.2-1.81) | 209.94 | 2.69(2.48-2.9) |
| Algeria | 0.4(0.09-0.78) | 0.56(0.13-1.1) | 1.74(0.41-3.37) | 0.87(0.21-1.68) | 334.66 | 1.54(1.45-1.62) |
| American Samoa | 0 | 0.79(0.17-1.48) | 0(0-0.01) | 1.22(0.27-2.33) | 196 | 1.59(1.45-1.73) |
| Andorra | 0(0-0.01) | 1.07(0.23-2.11) | 0.01(0-0.01) | 1.13(0.26-2.24) | 147.69 | 0.03(−0.08-0.14) |
| Angola | 0.04(0.01-0.08) | 0.15(0.03-0.31) | 0.22(0.05-0.45) | 0.26(0.06-0.52) | 472.47 | 2.12(2.05-2.19) |
| Antigua and Barbuda | 0 | 0.39(0.09-0.72) | 0(0-0.01) | 0.57(0.12-1.11) | 212.19 | 1.27(1.11-1.42) |
| Argentina | 1.57(0.36-2.89) | 0.9(0.21-1.68) | 2.62(0.55-5.03) | 0.94(0.2-1.82) | 67.56 | −0.23(−0.39-−0.07) |
| Armenia | 0.25(0.06-0.45) | 1.53(0.35-2.79) | 0.33(0.07-0.61) | 1.51(0.34-2.82) | 32.48 | −0.53(−0.81-−0.25) |
| Australia | 1.02(0.23-1.93) | 1.07(0.24-2.03) | 1.53(0.33-2.99) | 0.87(0.19-1.73) | 49.89 | −1.04(−1.19-−0.89) |
| Austria | 0.61(0.13-1.15) | 1.17(0.25-2.19) | 0.68(0.15-1.29) | 0.94(0.21-1.8) | 10.86 | −1.05(−1.19-−0.91) |
| Azerbaijan | 0.23(0.05-0.43) | 0.75(0.16-1.41) | 0.55(0.13-1.05) | 0.91(0.2-1.7) | 144.3 | 0.55(0.35-0.74) |
| Bahamas | 0.01(0-0.01) | 0.76(0.17-1.45) | 0.02(0-0.04) | 0.8(0.19-1.56) | 145.91 | 0.29(0.15-0.43) |
| Bahrain | 0.01(0-0.01) | 0.69(0.15-1.28) | 0.05(0.01-0.1) | 0.94(0.21-1.75) | 604.7 | 1(0.85-1.16) |
| Bangladesh | 1.12(0.22-2.16) | 0.42(0.08-0.82) | 4.23(0.93-8.06) | 0.59(0.13-1.12) | 277.36 | 0.7(0.58-0.83) |
| Barbados | 0.01(0-0.01) | 0.55(0.12-1.06) | 0.02(0-0.03) | 0.68(0.15-1.31) | 115.64 | 0.86(0.74-0.97) |
| Belarus | 0.6(0.14-1.13) | 0.87(0.2-1.64) | 0.77(0.17-1.5) | 0.94(0.21-1.85) | 26.91 | −0.24(−0.41-−0.07) |
| Belgium | 1(0.22-1.9) | 1.45(0.32-2.75) | 0.95(0.21-1.84) | 1.05(0.23-2.03) | −4.25 | −1.45(−1.61-−1.29) |
| Belize | 0 | 0.2(0.05-0.39) | 0.01(0-0.01) | 0.3(0.07-0.57) | 420.72 | 1.38(1.14-1.62) |
| Benin | 0.02(0-0.04) | 0.16(0.03-0.3) | 0.06(0.01-0.12) | 0.18(0.04-0.37) | 226.68 | 0.41(0.34-0.48) |
| Bermuda | 0(0-0.01) | 0.89(0.21-1.67) | 0(0-0.01) | 0.75(0.16-1.47) | 38.78 | −1.1(−1.27-−0.93) |
| Bhutan | 0 | 0.14(0.03-0.3) | 0.01(0-0.01) | 0.19(0.04-0.39) | 161.34 | 0.73(0.55-0.91) |
| Bolivia | 0.04(0.01-0.07) | 0.18(0.04-0.35) | 0.13(0.03-0.25) | 0.25(0.06-0.5) | 251.6 | 0.85(0.69-1.01) |
| Bosnia and Herzegovina | 0.12(0.03-0.22) | 0.48(0.11-0.9) | 0.3(0.07-0.58) | 1.03(0.24-2.01) | 153.82 | 3.43(3.13-3.73) |
| Botswana | 0.02(0-0.03) | 0.49(0.11-0.98) | 0.09(0.02-0.19) | 0.95(0.22-1.95) | 411.69 | 2.38(2.24-2.51) |
| Brazil | 2.49(0.59-4.56) | 0.47(0.11-0.85) | 5.6(1.27-10.54) | 0.43(0.1-0.8) | 125.07 | −0.42(−0.65-−0.2) |
| Brunei Darussalam | 0.01(0-0.01) | 0.8(0.17-1.55) | 0.02(0-0.04) | 0.93(0.2-1.76) | 240.94 | 1.02(0.66-1.39) |
| Bulgaria | 0.66(0.15-1.21) | 1.09(0.25-1.99) | 0.87(0.19-1.69) | 1.39(0.3-2.74) | 30.47 | 1.76(1.41-2.1) |
| Burkina Faso | 0.07(0.01-0.13) | 0.25(0.05-0.5) | 0.2(0.04-0.38) | 0.31(0.07-0.6) | 190.69 | 0.54(0.44-0.65) |
| Burundi | 0.02(0-0.05) | 0.15(0.03-0.3) | 0.05(0.01-0.1) | 0.15(0.03-0.31) | 93.13 | −0.42(−0.56-−0.28) |
| Cabo Verde | 0 | 0.21(0.05-0.41) | 0.01(0-0.01) | 0.29(0.07-0.55) | 193.95 | 1.38(1.03-1.72) |
| Cambodia | 0.11(0.02-0.22) | 0.34(0.07-0.66) | 0.43(0.1-0.83) | 0.55(0.12-1.07) | 291.41 | 1.6(1.52-1.68) |
| Cameroon | 0.06(0.01-0.12) | 0.21(0.04-0.41) | 0.23(0.05-0.47) | 0.28(0.06-0.57) | 293.51 | 0.94(0.87-1.02) |
| Canada | 1.86(0.42-3.48) | 1.15(0.26-2.19) | 2.27(0.48-4.38) | 0.79(0.16-1.54) | 22.15 | −1.8(−1.98-−1.62) |
| Central African Republic | 0.01(0-0.03) | 0.18(0.04-0.36) | 0.03(0.01-0.06) | 0.18(0.04-0.41) | 96.2 | 0.02(−0.01-0.06) |
| Chad | 0.02(0.01-0.05) | 0.15(0.03-0.29) | 0.07(0.02-0.14) | 0.2(0.05-0.39) | 182.15 | 1.11(1.08-1.15) |
| Chile | 0.37(0.08-0.68) | 0.65(0.14-1.2) | 0.89(0.2-1.72) | 0.71(0.16-1.39) | 141.77 | 0.5(0.41-0.59) |
| China | 24.5(5.17-45.84) | 0.51(0.11-0.96) | 101(22.13-186.92) | 0.98(0.21-1.82) | 312.3 | 2.38(2.3-2.46) |
| Colombia | 0.38(0.09-0.69) | 0.35(0.08-0.65) | 1.17(0.25-2.26) | 0.42(0.09-0.81) | 211.7 | 0.49(0.39-0.59) |
| Comoros | 0(0-0.01) | 0.25(0.04-0.51) | 0.01(0-0.02) | 0.38(0.08-0.76) | 241.24 | 1.22(1.05-1.39) |
| Congo | 0.02(0-0.03) | 0.23(0.05-0.48) | 0.06(0.01-0.14) | 0.35(0.07-0.74) | 300.48 | 1.56(1.42-1.71) |
| Cook Islands | 0 | 2.09(0.48-3.98) | 0(0-0.01) | 2.35(0.55-4.48) | 92.36 | 0.45(0.29-0.61) |
| Costa Rica | 0.06(0.01-0.11) | 0.62(0.14-1.16) | 0.17(0.04-0.34) | 0.62(0.13-1.22) | 178.52 | −0.12(−0.23-0) |
| Croatia | 0.5(0.12-0.93) | 1.45(0.34-2.72) | 0.52(0.12-1.01) | 1.31(0.3-2.52) | 5.25 | 0.28(0.03-0.52) |
| Cuba | 0.4(0.09-0.73) | 0.76(0.17-1.4) | 0.61(0.13-1.18) | 0.67(0.15-1.29) | 54.94 | −0.58(−0.64-−0.51) |
| Cyprus | 0.05(0.01-0.1) | 1.25(0.29-2.32) | 0.17(0.04-0.34) | 1.86(0.39-3.64) | 235.48 | 1.58(1.24-1.92) |
| Czechia | 0.68(0.15-1.25) | 0.99(0.22-1.82) | 0.71(0.16-1.36) | 0.81(0.18-1.56) | 4.87 | −1.06(−1.24-−0.87) |
| Côte d'Ivoire | 0.05(0.01-0.1) | 0.19(0.04-0.37) | 0.19(0.04-0.38) | 0.28(0.06-0.54) | 287.23 | 1.49(1.38-1.6) |
| Democratic People's Republic of Korea | 0.63(0.12-1.23) | 0.58(0.12-1.14) | 1.22(0.28-2.49) | 0.7(0.16-1.44) | 95.6 | 0.63(0.49-0.76) |
| Democratic Republic of the Congo | 0.13(0.03-0.26) | 0.13(0.03-0.25) | 0.41(0.09-0.83) | 0.17(0.04-0.34) | 210.57 | 0.77(0.58-0.97) |
| Denmark | 0.48(0.11-0.89) | 1.39(0.32-2.59) | 0.37(0.08-0.72) | 0.83(0.18-1.61) | −22.79 | −2.05(−2.19-−1.9) |
| Djibouti | 0(0-0.01) | 0.31(0.07-0.59) | 0.02(0-0.04) | 0.57(0.13-1.13) | 635.95 | 2.48(2.3-2.67) |
| Dominica | 0 | 0.49(0.11-0.97) | 0 | 0.52(0.12-1) | 28.32 | 0.2(0.15-0.25) |
| Dominican Republic | 0.04(0.01-0.07) | 0.17(0.04-0.31) | 0.14(0.03-0.27) | 0.27(0.06-0.53) | 277.35 | 1.91(1.71-2.12) |
| Ecuador | 0.04(0.01-0.08) | 0.14(0.03-0.26) | 0.21(0.05-0.39) | 0.25(0.05-0.47) | 365.63 | 1.93(1.83-2.04) |
| Egypt | 0.68(0.16-1.26) | 0.38(0.09-0.69) | 2.81(0.61-5.52) | 0.73(0.16-1.43) | 313.45 | 2.44(1.98-2.89) |
| El Salvador | 0.02(0-0.03) | 0.09(0.02-0.17) | 0.07(0.01-0.15) | 0.21(0.04-0.44) | 354.21 | 3.12(2.88-3.37) |
| Equatorial Guinea | 0 | 0.16(0.04-0.32) | 0.01(0-0.03) | 0.39(0.08-0.84) | 551.27 | 3.57(3.36-3.77) |
| Eritrea | 0.01(0-0.02) | 0.1(0.02-0.22) | 0.04(0.01-0.07) | 0.18(0.04-0.36) | 350.85 | 2(1.88-2.13) |
| Estonia | 0.1(0.02-0.19) | 0.91(0.22-1.67) | 0.1(0.02-0.2) | 0.91(0.2-1.78) | 0.33 | −0.25(−0.45-−0.05) |
| Eswatini | 0(0-0.01) | 0.24(0.05-0.46) | 0.01(0-0.02) | 0.29(0.06-0.6) | 148.83 | 1.07(0.78-1.37) |
| Ethiopia | 0.14(0.03-0.29) | 0.11(0.02-0.22) | 0.31(0.07-0.6) | 0.11(0.03-0.22) | 117.11 | −0.3(−0.58-−0.02) |
| Fiji | 0.02(0-0.05) | 0.98(0.2-1.91) | 0.05(0.01-0.1) | 1.2(0.26-2.34) | 118.14 | 0.95(0.71-1.19) |
| Finland | 0.3(0.07-0.55) | 0.9(0.2-1.65) | 0.35(0.08-0.66) | 0.79(0.18-1.56) | 14 | −0.6(−0.84-−0.37) |
| France | 4.49(0.98-8.35) | 1.25(0.27-2.32) | 4.93(1.06-9.57) | 0.99(0.21-1.92) | 9.88 | −1.15(−1.44-−0.86) |
| Gabon | 0.01(0-0.02) | 0.29(0.06-0.56) | 0.03(0.01-0.06) | 0.41(0.09-0.83) | 199.09 | 1.06(0.88-1.24) |
| Gambia | 0(0-0.01) | 0.16(0.04-0.32) | 0.02(0-0.03) | 0.27(0.06-0.55) | 382.78 | 1.56(1.36-1.75) |
| Georgia | 0.5(0.12-0.91) | 1.48(0.35-2.71) | 0.43(0.1-0.8) | 1.54(0.35-2.84) | −12.68 | 0.36(0.15-0.57) |
| Germany | 5.25(1.15-9.58) | 0.91(0.2-1.65) | 6.28(1.39-12.48) | 0.92(0.21-1.83) | 19.61 | −0.13(−0.32-0.06) |
| Ghana | 0.08(0.02-0.16) | 0.19(0.04-0.37) | 0.33(0.07-0.67) | 0.29(0.06-0.58) | 312.18 | 1.14(1.04-1.24) |
| Greece | 1.14(0.25-2.1) | 1.58(0.35-2.93) | 1.42(0.31-2.8) | 1.53(0.33-3.01) | 24.92 | −0.1(−0.24-0.05) |
| Greenland | 0 | 1.03(0.23-1.93) | 0 | 0.7(0.15-1.34) | 14.34 | −1.92(−2.13-−1.71) |
| Grenada | 0 | 0.45(0.1-0.85) | 0(0-0.01) | 0.63(0.14-1.18) | 139.92 | 1.24(1.02-1.47) |
| Guam | 0(0-0.01) | 0.77(0.17-1.42) | 0.01(0-0.02) | 0.89(0.2-1.65) | 137.01 | 0.24(0.03-0.46) |
| Guatemala | 0.03(0.01-0.05) | 0.11(0.03-0.22) | 0.13(0.03-0.26) | 0.2(0.04-0.38) | 422.24 | 2.25(2.07-2.43) |
| Guinea | 0.04(0.01-0.07) | 0.2(0.04-0.39) | 0.09(0.02-0.18) | 0.26(0.06-0.53) | 133.85 | 0.96(0.92-1) |
| Guinea-Bissau | 0.01(0-0.01) | 0.22(0.05-0.44) | 0.02(0-0.03) | 0.31(0.07-0.64) | 172.77 | 1.25(1.19-1.32) |
| Guyana | 0.01(0-0.02) | 0.47(0.1-0.88) | 0.02(0-0.04) | 0.54(0.12-1.03) | 82.93 | 0.27(0.01-0.54) |
| Haiti | 0.05(0.01-0.11) | 0.26(0.06-0.52) | 0.12(0.03-0.25) | 0.26(0.06-0.53) | 132.43 | −0.07(−0.24-0.1) |
| Honduras | 0.02(0-0.05) | 0.19(0.04-0.37) | 0.12(0.03-0.24) | 0.32(0.07-0.65) | 388.67 | 1.72(1.61-1.82) |
| Hungary | 0.87(0.2-1.6) | 1.15(0.26-2.13) | 1.01(0.22-1.91) | 1.13(0.25-2.14) | 16.33 | −0.38(−0.67-−0.08) |
| Iceland | 0.02(0-0.04) | 1.42(0.3-2.72) | 0.02(0-0.03) | 0.74(0.16-1.39) | −7.92 | −2.56(−2.7-−2.42) |
| India | 7.11(1.62-13.34) | 0.27(0.06-0.5) | 24.93(5.84-45.88) | 0.39(0.09-0.72) | 250.69 | 1.24(1.01-1.46) |
| Indonesia | 4.61(1.08-8.55) | 0.67(0.16-1.24) | 15.13(3.51-29.3) | 1.09(0.25-2.09) | 227.96 | 1.65(1.55-1.76) |
| Iran  (Islamic Republic of) | 0.57(0.13-1.06) | 0.36(0.08-0.68) | 3.09(0.72-5.79) | 0.69(0.16-1.29) | 444.45 | 2.1(1.93-2.27) |
| Iraq | 0.29(0.06-0.59) | 0.63(0.14-1.25) | 1.8(0.39-3.52) | 1.17(0.25-2.27) | 515.31 | 2.26(2.14-2.37) |
| Ireland | 0.29(0.06-0.53) | 1.54(0.35-2.82) | 0.38(0.08-0.73) | 1.11(0.23-2.1) | 32.46 | −1.37(−1.52-−1.22) |
| Israel | 0.29(0.06-0.54) | 1.22(0.25-2.31) | 0.55(0.12-1.09) | 1.06(0.24-2.13) | 92.76 | −0.82(−1-−0.64) |
| Italy | 6.68(1.46-12.33) | 1.68(0.37-3.07) | 7.25(1.61-13.88) | 1.39(0.3-2.7) | 8.6 | −0.71(−0.87-−0.55) |
| Jamaica | 0.05(0.01-0.09) | 0.53(0.13-0.98) | 0.13(0.03-0.25) | 0.86(0.19-1.68) | 185.29 | 1.6(1.29-1.91) |
| Japan | 7.34(1.6-13.65) | 0.85(0.19-1.58) | 11.7(2.7-22.54) | 1.08(0.24-2.11) | 59.39 | 0.92(0.78-1.06) |
| Jordan | 0.08(0.02-0.15) | 0.91(0.2-1.72) | 0.53(0.11-1.05) | 1.3(0.28-2.52) | 577.34 | 1.17(0.92-1.43) |
| Kazakhstan | 0.61(0.14-1.11) | 0.79(0.18-1.41) | 0.88(0.2-1.65) | 0.83(0.18-1.54) | 44.01 | 0.58(0.42-0.74) |
| Kenya | 0.09(0.02-0.19) | 0.17(0.03-0.35) | 0.33(0.08-0.64) | 0.22(0.05-0.42) | 282.7 | 0.6(0.48-0.72) |
| Kiribati | 0 | 0.9(0.21-1.69) | 0.01(0-0.01) | 1.11(0.26-2.18) | 140.84 | 0.62(0.48-0.76) |
| Kuwait | 0.04(0.01-0.07) | 0.96(0.23-1.81) | 0.18(0.04-0.35) | 1(0.23-1.95) | 404.95 | 0.18(−0.15-0.52) |
| Kyrgyzstan | 0.12(0.03-0.22) | 0.69(0.16-1.28) | 0.15(0.03-0.29) | 0.53(0.12-0.97) | 28.24 | −1.21(−1.59-−0.83) |
| Lao People's Democratic Republic | 0.08(0.02-0.17) | 0.63(0.13-1.3) | 0.22(0.05-0.45) | 0.77(0.17-1.57) | 167.78 | 0.55(0.48-0.63) |
| Latvia | 0.18(0.04-0.33) | 0.92(0.21-1.68) | 0.19(0.04-0.37) | 1.05(0.23-2.03) | 6.33 | 0.44(0.3-0.59) |
| Lebanon | 0.12(0.03-0.23) | 0.92(0.21-1.76) | 0.74(0.17-1.42) | 2.58(0.57-4.94) | 526.79 | 4.16(3.92-4.4) |
| Lesotho | 0.01(0-0.03) | 0.26(0.06-0.52) | 0.05(0.01-0.11) | 0.63(0.14-1.33) | 246.04 | 4.33(3.89-4.77) |
| Liberia | 0.01(0-0.02) | 0.17(0.04-0.33) | 0.03(0.01-0.06) | 0.22(0.04-0.47) | 210.43 | 1.08(0.91-1.24) |
| Libya | 0.05(0.01-0.1) | 0.49(0.11-0.94) | 0.35(0.07-0.69) | 1.04(0.22-2.04) | 604.62 | 2.88(2.6-3.17) |
| Lithuania | 0.22(0.05-0.4) | 0.91(0.2-1.67) | 0.2(0.04-0.39) | 0.82(0.18-1.59) | −6.63 | −0.25(−0.33-−0.17) |
| Luxembourg | 0.04(0.01-0.07) | 1.42(0.31-2.6) | 0.05(0.01-0.09) | 1.05(0.23-2.01) | 28.12 | −0.93(−1.22-−0.64) |
| Madagascar | 0.09(0.02-0.17) | 0.27(0.06-0.54) | 0.21(0.05-0.42) | 0.27(0.06-0.52) | 143.76 | −0.32(−0.45-−0.2) |
| Malawi | 0.04(0.01-0.09) | 0.18(0.04-0.33) | 0.1(0.02-0.2) | 0.2(0.05-0.4) | 123.77 | 0.44(0.33-0.55) |
| Malaysia | 0.5(0.11-0.94) | 0.86(0.18-1.61) | 2.26(0.51-4.33) | 1.52(0.34-2.91) | 350.44 | 2.01(1.88-2.14) |
| Maldives | 0(0-0.01) | 0.64(0.12-1.33) | 0.02(0-0.03) | 0.93(0.22-1.75) | 376.22 | 1.03(0.81-1.25) |
| Mali | 0.04(0.01-0.08) | 0.18(0.05-0.34) | 0.13(0.03-0.26) | 0.25(0.05-0.48) | 192.9 | 0.9(0.74-1.06) |
| Malta | 0.03(0.01-0.06) | 1.31(0.3-2.47) | 0.03(0.01-0.06) | 0.98(0.21-1.86) | 13.57 | −0.86(−0.95-−0.77) |
| Marshall Islands | 0 | 0.73(0.17-1.38) | 0(0-0.01) | 1.21(0.24-2.48) | 272.38 | 1.64(1.46-1.82) |
| Mauritania | 0.01(0-0.03) | 0.25(0.05-0.5) | 0.04(0.01-0.07) | 0.3(0.06-0.59) | 160.01 | 0.69(0.6-0.77) |
| Mauritius | 0.03(0.01-0.05) | 0.59(0.13-1.1) | 0.12(0.03-0.23) | 1.33(0.29-2.57) | 345.9 | 2.85(2.71-2.99) |
| Mexico | 1.04(0.24-1.89) | 0.4(0.09-0.72) | 3.07(0.7-6.21) | 0.46(0.1-0.92) | 195.56 | −0.14(−0.36-0.08) |
| Micronesia  (Federated States of) | 0 | 0.88(0.19-1.66) | 0.01(0-0.01) | 1.5(0.3-3.23) | 177.86 | 1.92(1.84-2.01) |
| Monaco | 0(0-0.01) | 1.61(0.35-3.03) | 0.01(0-0.01) | 1.87(0.39-3.76) | 45.39 | 0.77(0.57-0.97) |
| Mongolia | 0.02(0-0.03) | 0.29(0.07-0.56) | 0.07(0.01-0.13) | 0.4(0.08-0.78) | 276.74 | 0.82(0.64-1) |
| Montenegro | 0.03(0.01-0.06) | 0.87(0.2-1.63) | 0.07(0.02-0.14) | 1.49(0.34-2.85) | 140.07 | 2.34(2.04-2.65) |
| Morocco | 0.47(0.11-0.9) | 0.56(0.13-1.07) | 1.74(0.38-3.5) | 0.93(0.2-1.86) | 267.96 | 1.62(1.57-1.67) |
| Mozambique | 0.06(0.01-0.11) | 0.15(0.03-0.29) | 0.21(0.04-0.42) | 0.27(0.05-0.54) | 258.16 | 2.43(2.21-2.65) |
| Myanmar | 1.57(0.33-3.2) | 1.06(0.22-2.13) | 1.65(0.38-3.2) | 0.57(0.13-1.1) | 4.96 | −2.46(−2.71-−2.21) |
| Namibia | 0.01(0-0.03) | 0.33(0.08-0.63) | 0.06(0.01-0.12) | 0.68(0.15-1.37) | 353.5 | 2.9(2.8-3) |
| Nauru | 0 | 1.28(0.27-2.5) | 0 | 1.95(0.41-3.92) | 99.96 | 1.37(1.26-1.47) |
| Nepal | 0.14(0.03-0.29) | 0.25(0.05-0.51) | 0.52(0.11-1.02) | 0.4(0.09-0.78) | 259.56 | 1.45(1.35-1.55) |
| Netherlands | 1.62(0.35-3.05) | 1.74(0.38-3.28) | 1.73(0.36-3.29) | 1.29(0.27-2.5) | 6.63 | −1.37(−1.53-−1.21) |
| New Zealand | 0.26(0.06-0.49) | 1.4(0.31-2.62) | 0.37(0.08-0.72) | 1.13(0.24-2.2) | 43.63 | −0.73(−0.82-−0.64) |
| Nicaragua | 0.02(0-0.03) | 0.18(0.04-0.34) | 0.1(0.02-0.2) | 0.38(0.08-0.73) | 480.5 | 2.72(2.6-2.83) |
| Niger | 0.02(0-0.04) | 0.11(0.02-0.21) | 0.07(0.02-0.14) | 0.13(0.03-0.28) | 258.04 | 0.63(0.52-0.74) |
| Nigeria | 0.43(0.09-0.88) | 0.19(0.04-0.38) | 1.51(0.3-3.09) | 0.25(0.05-0.5) | 251.79 | 1.08(0.94-1.22) |
| Niue | 0 | 1.12(0.25-2.19) | 0 | 1.77(0.4-3.53) | 49.86 | 1.58(1.42-1.74) |
| North Macedonia | 0.1(0.02-0.18) | 0.94(0.21-1.77) | 0.23(0.05-0.44) | 1.43(0.33-2.75) | 131.39 | 1.56(1.34-1.77) |
| Northern Mariana Islands | 0 | 1.47(0.31-2.85) | 0(0-0.01) | 1.53(0.35-3) | 126.64 | −0.11(−0.26-0.03) |
| Norway | 0.29(0.07-0.54) | 1.05(0.24-1.96) | 0.29(0.06-0.56) | 0.74(0.17-1.47) | −2.58 | −1.48(−1.81-−1.15) |
| Oman | 0.01(0-0.02) | 0.28(0.06-0.57) | 0.06(0.01-0.11) | 0.58(0.13-1.12) | 494.73 | 2.74(2.39-3.09) |
| Pakistan | 2.41(0.55-4.85) | 0.8(0.18-1.61) | 8.65(1.86-17.2) | 1.27(0.27-2.47) | 259.08 | 1.28(1.09-1.46) |
| Palau | 0 | 1.48(0.29-2.94) | 0 | 1.98(0.44-3.86) | 166.52 | 0.97(0.88-1.07) |
| Palestine | 0.05(0.01-0.09) | 0.89(0.19-1.75) | 0.23(0.05-0.44) | 1.46(0.33-2.77) | 377.26 | 1.64(1.35-1.93) |
| Panama | 0.02(0.01-0.04) | 0.28(0.06-0.53) | 0.06(0.01-0.12) | 0.29(0.06-0.56) | 160.38 | −0.28(−0.55-−0.01) |
| Papua New Guinea | 0.15(0.04-0.3) | 1.28(0.29-2.49) | 0.54(0.12-1.05) | 1.61(0.37-3.13) | 249.44 | 0.72(0.63-0.82) |
| Paraguay | 0.04(0.01-0.08) | 0.34(0.08-0.63) | 0.17(0.04-0.34) | 0.56(0.12-1.11) | 296.49 | 1.48(1.33-1.62) |
| Peru | 0.14(0.03-0.27) | 0.2(0.05-0.38) | 0.4(0.09-0.79) | 0.23(0.05-0.45) | 187.44 | −0.21(−0.47-0.06) |
| Philippines | 1.57(0.37-2.85) | 0.83(0.2-1.49) | 4.42(0.94-8.61) | 0.92(0.2-1.79) | 182.33 | 0.01(−0.22-0.24) |
| Poland | 1.67(0.37-3.02) | 0.72(0.16-1.33) | 2.49(0.54-4.79) | 0.78(0.17-1.51) | 48.99 | 0.34(0.19-0.49) |
| Portugal | 0.95(0.2-1.72) | 1.44(0.31-2.64) | 1.17(0.25-2.25) | 1.25(0.27-2.43) | 23.96 | −0.77(−1.01-−0.53) |
| Puerto Rico | 0.1(0.02-0.18) | 0.51(0.11-0.94) | 0.16(0.04-0.31) | 0.57(0.13-1.11) | 65.76 | 0.19(0.1-0.29) |
| Qatar | 0.01(0-0.01) | 0.89(0.21-1.73) | 0.08(0.02-0.16) | 1.74(0.38-3.32) | 1210.03 | 2.88(2.6-3.16) |
| Republic of Korea | 1.02(0.23-1.89) | 0.51(0.11-0.95) | 3.42(0.77-6.45) | 0.81(0.18-1.53) | 235.81 | 1.64(1.43-1.84) |
| Republic of Moldova | 0.2(0.04-0.36) | 0.78(0.18-1.42) | 0.18(0.04-0.33) | 0.61(0.13-1.13) | −8.6 | −0.55(−0.72-−0.37) |
| Romania | 0.92(0.2-1.7) | 0.64(0.14-1.19) | 1.42(0.31-2.69) | 0.87(0.19-1.66) | 54.83 | 0.93(0.76-1.11) |
| Russian Federation | 8.23(1.89-14.94) | 0.82(0.19-1.47) | 12.92(2.92-24.65) | 1.08(0.25-2.03) | 56.98 | 0.75(0.51-0.99) |
| Rwanda | 0.05(0.01-0.09) | 0.24(0.05-0.47) | 0.1(0.02-0.2) | 0.24(0.05-0.46) | 123.3 | −0.5(−0.86-−0.13) |
| Saint Kitts and Nevis | 0 | 1.15(0.25-2.18) | 0(0-0.01) | 0.78(0.17-1.56) | 46.46 | −1.54(−1.66-−1.41) |
| Saint Lucia | 0 | 0.48(0.11-0.87) | 0.01(0-0.01) | 0.45(0.1-0.86) | 125.48 | −0.44(−0.66-−0.22) |
| Saint Vincent and the Grenadines | 0 | 0.56(0.13-1.08) | 0(0-0.01) | 0.64(0.14-1.22) | 101.38 | 0.22(0.03-0.41) |
| Samoa | 0(0-0.01) | 0.85(0.19-1.65) | 0.01(0-0.02) | 1.1(0.23-2.43) | 119.68 | 0.76(0.67-0.85) |
| San Marino | 0 | 0.97(0.22-1.83) | 0(0-0.01) | 1.07(0.23-2.08) | 88.71 | 0.65(0.53-0.77) |
| Sao Tome and Principe | 0 | 0.09(0.02-0.18) | 0 | 0.17(0.03-0.36) | 293.83 | 2.07(1.92-2.22) |
| Saudi Arabia | 0.09(0.02-0.17) | 0.27(0.06-0.53) | 1.18(0.25-2.3) | 0.88(0.19-1.71) | 1242.83 | 4.25(4.12-4.38) |
| Senegal | 0.07(0.02-0.14) | 0.38(0.09-0.72) | 0.19(0.04-0.39) | 0.42(0.09-0.84) | 169.2 | 0.17(0.02-0.32) |
| Serbia | 0.56(0.13-1.05) | 0.93(0.21-1.75) | 1.02(0.23-1.99) | 1.4(0.32-2.75) | 80.99 | 1.25(1.03-1.47) |
| Seychelles | 0 | 0.57(0.12-1.07) | 0.01(0-0.01) | 1.19(0.27-2.32) | 333.11 | 2.62(2.38-2.86) |
| Sierra Leone | 0.03(0.01-0.05) | 0.26(0.06-0.51) | 0.09(0.02-0.18) | 0.4(0.09-0.82) | 226.74 | 1.68(1.56-1.8) |
| Singapore | 0.09(0.02-0.18) | 0.61(0.13-1.18) | 0.28(0.06-0.55) | 0.71(0.16-1.38) | 213.32 | 0.75(0.51-0.98) |
| Slovakia | 0.28(0.06-0.51) | 0.9(0.2-1.65) | 0.42(0.1-0.83) | 0.93(0.21-1.84) | 49.7 | 0.11(0-0.22) |
| Slovenia | 0.14(0.03-0.26) | 1.09(0.23-2.07) | 0.15(0.03-0.28) | 0.85(0.19-1.63) | 6.58 | −0.71(−0.78-−0.63) |
| Solomon Islands | 0(0-0.01) | 0.57(0.13-1.14) | 0.07(0.02-0.14) | 3.01(0.67-5.92) | 1424.03 | 6.18(5.66-6.7) |
| Somalia | 0.03(0.01-0.06) | 0.15(0.03-0.3) | 0.07(0.01-0.16) | 0.15(0.03-0.33) | 149.54 | 0.15(0.04-0.26) |
| South Africa | 0.78(0.19-1.42) | 0.58(0.14-1.07) | 1.38(0.3-2.56) | 0.5(0.11-0.93) | 78.2 | −0.28(−0.41-−0.15) |
| South Sudan | 0.02(0-0.04) | 0.15(0.03-0.3) | 0.04(0.01-0.08) | 0.16(0.04-0.32) | 105.33 | 0.17(0.03-0.3) |
| Spain | 2.97(0.67-5.53) | 1.21(0.27-2.24) | 4.05(0.89-7.84) | 1.06(0.23-2.07) | 36.33 | −0.58(−0.71-−0.45) |
| Sri Lanka | 0.22(0.05-0.42) | 0.34(0.07-0.63) | 0.86(0.19-1.71) | 0.63(0.14-1.24) | 285.81 | 2.63(2.45-2.81) |
| Sudan | 0.09(0.02-0.18) | 0.17(0.03-0.35) | 0.38(0.08-0.81) | 0.31(0.07-0.63) | 321.74 | 2.1(1.92-2.27) |
| Suriname | 0.01(0-0.01) | 0.42(0.1-0.8) | 0.02(0-0.04) | 0.61(0.13-1.14) | 222.46 | 1.21(1.04-1.37) |
| Sweden | 0.44(0.1-0.84) | 0.76(0.16-1.43) | 0.45(0.09-0.85) | 0.6(0.13-1.15) | 0.29 | −0.95(−1.1-−0.81) |
| Switzerland | 0.71(0.16-1.33) | 1.51(0.33-2.82) | 0.67(0.15-1.28) | 0.94(0.22-1.82) | −5.87 | −2(−2.16-−1.84) |
| Syrian Arab Republic | 0.1(0.02-0.2) | 0.33(0.07-0.64) | 0.42(0.09-0.84) | 0.57(0.12-1.12) | 303.03 | 1.92(1.73-2.1) |
| Taiwan  (Province of China) | 0.6(0.13-1.09) | 0.67(0.15-1.23) | 2.14(0.46-4.05) | 1.15(0.25-2.2) | 257.96 | 1.75(1.32-2.2) |
| Tajikistan | 0.06(0.01-0.11) | 0.37(0.08-0.7) | 0.11(0.02-0.22) | 0.31(0.07-0.61) | 89.1 | −0.65(−0.89-−0.4) |
| Thailand | 1.09(0.23-2.07) | 0.47(0.1-0.89) | 3.74(0.82-7.52) | 0.71(0.16-1.43) | 243.95 | 1.43(0.99-1.87) |
| Timor-Leste | 0.01(0-0.02) | 0.42(0.1-0.83) | 0.03(0.01-0.05) | 0.59(0.12-1.17) | 205.6 | 1.37(1.01-1.72) |
| Togo | 0.02(0-0.03) | 0.19(0.04-0.36) | 0.06(0.01-0.12) | 0.21(0.05-0.44) | 252.1 | 0.3(0.12-0.47) |
| Tokelau | 0 | 1(0.22-2.01) | 0 | 1.63(0.35-3.27) | 59.97 | 1.75(1.68-1.83) |
| Tonga | 0(0-0.01) | 1.1(0.23-2.06) | 0.01(0-0.01) | 1.35(0.32-2.61) | 65.44 | 0.43(0.3-0.55) |
| Trinidad and Tobago | 0.03(0.01-0.06) | 0.69(0.16-1.28) | 0.07(0.02-0.15) | 0.8(0.18-1.6) | 131.86 | 0.64(0.44-0.84) |
| Tunisia | 0.18(0.04-0.33) | 0.66(0.14-1.2) | 0.78(0.18-1.51) | 1.13(0.26-2.19) | 324.43 | 1.83(1.7-1.96) |
| Turkey | 1.07(0.24-2.04) | 0.51(0.11-0.97) | 3.5(0.75-6.63) | 0.73(0.16-1.39) | 227.73 | 1.59(1.34-1.83) |
| Turkmenistan | 0.06(0.01-0.12) | 0.52(0.11-0.97) | 0.17(0.04-0.32) | 0.66(0.14-1.28) | 162.87 | 1.06(0.59-1.53) |
| Tuvalu | 0 | 1.01(0.23-1.93) | 0 | 1.42(0.32-2.84) | 80.11 | 1.09(0.99-1.19) |
| Uganda | 0.07(0.01-0.13) | 0.16(0.04-0.33) | 0.28(0.06-0.56) | 0.26(0.06-0.54) | 314.9 | 1.18(0.87-1.49) |
| Ukraine | 3.93(0.84-7.26) | 1.04(0.23-1.9) | 3.3(0.7-6.51) | 0.88(0.18-1.77) | −15.99 | −1.1(−1.33-−0.88) |
| United Arab Emirates | 0.02(0-0.04) | 0.86(0.2-1.69) | 0.28(0.06-0.54) | 1.24(0.27-2.38) | 1351.36 | 1.17(0.71-1.62) |
| United Kingdom | 5.83(1.29-10.57) | 1.5(0.33-2.72) | 5.9(1.24-10.81) | 1.17(0.25-2.16) | 1.15 | −1.03(−1.09-−0.97) |
| United Republic of Tanzania | 0.13(0.03-0.25) | 0.2(0.05-0.38) | 0.46(0.1-0.89) | 0.29(0.06-0.56) | 249.17 | 1.3(1.2-1.4) |
| United States of America | 19.13(4.11-35.33) | 1.26(0.27-2.33) | 18.86(3.78-36.06) | 0.77(0.15-1.47) | −1.4 | −2.32(−2.52-−2.12) |
| United States Virgin Islands | 0(0-0.01) | 0.54(0.12-1.03) | 0(0-0.01) | 0.57(0.13-1.11) | 72.92 | 0.09(−0.05-0.24) |
| Uruguay | 0.27(0.06-0.5) | 1.37(0.31-2.55) | 0.3(0.07-0.58) | 1.17(0.26-2.26) | 13.17 | −0.79(−0.91-−0.67) |
| Uzbekistan | 0.28(0.07-0.52) | 0.42(0.1-0.78) | 0.9(0.2-1.71) | 0.59(0.13-1.12) | 221.01 | 0.82(0.64-1.01) |
| Vanuatu | 0 | 0.49(0.12-0.98) | 0.01(0-0.02) | 0.77(0.16-1.59) | 315.87 | 1.27(0.99-1.55) |
| Venezuela | 0.26(0.06-0.48) | 0.43(0.1-0.81) | 1.12(0.24-2.2) | 0.7(0.15-1.39) | 335.31 | 1.62(1.54-1.71) |
| Viet Nam | 1.75(0.38-3.36) | 0.74(0.16-1.42) | 8.04(1.68-15.62) | 1.4(0.3-2.71) | 358.91 | 2.55(2.4-2.7) |
| Yemen | 0.07(0.02-0.15) | 0.24(0.05-0.49) | 0.38(0.08-0.77) | 0.42(0.09-0.85) | 436.05 | 2.34(2.2-2.49) |
| Zambia | 0.06(0.01-0.11) | 0.3(0.06-0.6) | 0.19(0.04-0.38) | 0.39(0.08-0.78) | 237.7 | 0.76(0.66-0.86) |
| Zimbabwe | 0.09(0.02-0.18) | 0.37(0.08-0.7) | 0.27(0.06-0.55) | 0.55(0.12-1.1) | 195.48 | 2.1(1.78-2.42) |

EAPC: estimated annual percentage change; ASR, age-standardized rate; CI, confidence interval; UI: uncertainty interval; YLDs, years lived with disability.
